# Carotid artery plaque intervention with Tongxinluo capsule (CAPITAL): A multicenter randomized double-blind parallel-group placebo-controlled study

**DOI:** 10.1038/s41598-019-41118-z

**Published:** 2019-03-14

**Authors:** Mei Zhang, Yan Liu, Mingjun Xu, Lei Zhang, Yan Liu, Xiaoling Liu, Yuxia Zhao, Fang Zhu, Rui Xu, Zhihong Ou, Ying Wang, Qigong Liu, Shuping Ma, Tian Wang, Maolin He, Qinghua Lu, Honghua Li, Jihan Huang, Yun Zhang

**Affiliations:** 1 Department of Cardiology, Qilu Hospital of Shandong University, Jinan, China; 2 Department of Cardiology, People’s Hospital of Liaoning Province, Shen Yang, China; 3 Department of Cardiology, Shandong Provincial Qianfoshan Hospital, Jinan, China; 4 Department of Cardiology, People’s Hospital of Linyi City, Lin Yi, China; 5 Department of Cardiology, Second Affiliated Hospital of Shandong Traditional Chinese Medicine University, Jinan, China; 6 Department of Cardiology, Hospital Affiliated with Tongji Medical College of Huazhong University of Science and Technology, Wuhan, China; 7 Department of Cardiology, Hebei Provincial People’s Hospital, Shi Jiazhuang, China; 8 Department of Cardiology, Traffic Hospital of Shandong Province, Jinan, China; 9 Department of Cardiology, Beijing Shijitan Hospital, Beijing, China; 10 Department of Cardiology, Second Affiliated Hospital of Shandong University, Jinan, China; 11 Department of Cardiology, Wuhan General Hospital of Guangzhou Military Region, Wuhan, China; 12 Center for Drug Clinical Research, Shanghai University of Traditional Chinese Medicine, Shanghai, China

## Abstract

To determine whether the traditional Chinese medicine Tongxinluo (TXL) is efficacious at retarding the progression of carotid atherosclerotic lesions, a total of 1,212 patients with a focal intima-media thickness (IMT) of ≥1.2 mm of the carotid arteries received TXL or placebo capsules in addition to current routine therapy. The primary outcome was between-group differences in annualized change in mean IMT of 12 sites of bilateral carotid arteries over 24 months. The secondary outcomes were between-group differences in plaque area, vascular remodeling index (RI), serum levels of lipids and high-sensitivity C-reactive protein, and a composite of first major cardiovascular events. The results showed that the annualized change in mean IMT in the TXL and placebo groups was −0.00095 (95% CI, −0.00330 to 0.00141) mm and 0.01312 (95% CI, 0.01076 to 0.01548) mm, respectively, with a difference between the two groups of −0.01407 (95% CI, −0.01740 to −0.01073) mm (P < 0.001). Compared with placebo, TXL treatment significantly reduced the change from baseline in the plaque area and RI, as well as the first major cardiovascular events. In conclusion, TXL retarded the progression of mean IMT, plaque area and vascular remodeling of the carotid artery with a good safety profile.

## Introduction

In the last three decades, many clinical trials have demonstrated the efficacy of statins at reducing cardiovascular events for both primary and secondary prevention, making statins the cornerstone in the prevention and treatment of atherosclerosis. However, many patients still have high residual risk for cardiovascular events even after intensive statin treatment. In the study of the HPS2-THRIVE Collaborative Group, when added to statin-based LDL-lowering therapy, allocation to extended release niacin/laropiprant (ERN/LRPT) increased the risk of definite myopathy (75 (0.16%/year) vs. 17 (0.04%/year): risk ratio 4.4; 95% CI 2.6–7.5; P < 0.001), and the risk of myopathy was increased by adding ERN/LRPT to 40 mg daily simvastatin (with or without ezetimibe), particularly in Chinese patients whose myopathy rates on simvastatin were higher^1^. Therefore, exploration of new anti-atherosclerotic drugs with high efficacy and low toxicity is highly warranted.

Tongxinluo (TXL) capsule was approved in 1996 by the State Food and Drug Administration of China for treating angina pectoris and ischemic stroke, and it is extracted, concentrated, and freeze-dried from 7 plant and 5 animal products^2^. Recent experimental studies with hyperlipidemic rabbits and ApoE−/− mice revealed that TXL can attenuate and stabilize atherosclerotic plaques via its lipid-lowering, anti-oxidative and anti-inflammatory effects^3–5^. A few small clinical studies showed that TXL capsules lowered serum lipid levels, improved angina pectoris and reduced the incidence of restenosis in patients with coronary heart disease^6–8^. The ENLEAT trial showed that TXL treatment in addition to conventional medical therapy reduced the incidence of no-reflow and myocardial infarction area significantly after primary PCI in 219 patients with ST segment elevation myocardial infarction^9^. These basic and clinical studies strongly suggest that TXL might have a potent anti-atherosclerotic effect, but evidence from large-scale clinical trials of TXL therapy in patients with atherosclerosis is still lacking.

Since the pioneering work of O’Leary *et al*.^10^, many studies have confirmed the usefulness of carotid intima-media thickness (IMT) as a strong predictor of risk of myocardial infarction and stroke in subjects without a history of cardiovascular diseases^11,12^. In a recent meta-analysis of 8 studies with 37,197 subjects, the age- and sex-adjusted overall estimates of the relative risk of myocardial infarction and stroke were 1.26 and 1.32, respectively, per 1–standard deviation common carotid IMT difference^13^. Moreover, carotid IMT has been increasingly used as a surrogate outcome for evaluating the anti-atherosclerotic effects of various medications^14–17^. Although ultrasonographic measurement of the carotid IMT is useful, this variable is limited in predicting cardiovascular events. Compared with IMT, carotid plaque area may be more closely associated with atherosclerosis risk and cardiovascular events and has been recommended as another surrogate endpoint for atherosclerosis^18,19^.

Here, we report the results from the Carotid Artery Plaque Intervention with Tongxinluo CApsuLe (CAPITAL) trial designed to examine the effects of TXL capsules on carotid IMT and plaque area over 24 months in a large cohort of patients with subclinical atherosclerosis from China.

## Methods

### Design and eligibility

The CAPITAL study is a multicenter, randomized, double-blind, parallel-group, placebo-controlled clinical trial. The trial was designed and led by an executive steering committee, and the ethics approval was passed by the ethics committee of Qilu Hospital of Shandong University (see Supplementary Appendix I**)**. The study was conducted in accordance with the principles of Good Clinical Practice and the Declaration of Helsinki. This trial was registered with Chinese Clinical Trail Registry (http://WWW.chictr.org.cn) number ChiCTR-TRC-08000212 and the date of registration was December 5, 2008.

Subjects were initially screened for eligibility at baseline by detailed history-taking, physical examination, blood chemistry studies and electrocardiography and ultrasonography examinations. The inclusion criteria included men or women aged 40 to 75 years with non-calcified plaque defined as a focal thickening of the carotid IMT of 1.2 to 3.5 mm in bilateral common or internal carotid arteries detected by ultrasonography. The major exclusion criteria were a history of myocardial infarction and stroke within the past 6 months, a history of percutaneous coronary intervention or carotid endarterectomy, uncontrolled diabetes mellitus or hypertension, familial hypercholesterolemia, Takayasu arteritis, systemic disease, liver or renal dysfunction, pregnancy or lactation, and a requirement for warfarin for anticoagulant therapy or a history of receiving any other investigational traditional Chinese medicine within 12 months before the baseline visit. After the initial screening stage, informed consent was obtained from patients of 35 hospitals from 18 provinces in China who met all eligibility criteria; the patients were allocated to either TXL group or placebo group at a 1:1 ratio in permuted stacked blocks stratified by study center and then entered into a double-blind study with a scheduled duration of 24 months. The details of criteria for subject withdrawal and study termination are in Supplementary Appendix II.

### Drug administration

The TXL capsules used in this trial contained Ginseng radix et rhizoma (Araliaceae; Chinese ginseng), Paeoniaeradixrubra (Paeoniaceae; Chinese peony), Ziziphispinosae semen (Rhamnaceae; jujube seed) (fried), dalbergiaeodoriferae lignum (Fabaceae; Huanghuali wood), Santali albi lignum (Santalaceae; sandalwood), olibanum (Burseraceae; Boswellia)(prepared), Hirudo (Haemopidae, leech), Scorpio (Buthidae; Chinese scorpion), Scolopendra (Scolopendrasubspinipesmutilans L. Koch), Cicadae periostracum (Cicadidae; cicada), EupolyphagaSteleophaga (Corydiidae; Woodlouse), and Borneolum (Borneolumsyntheticum). The criteria for the quality of the herbs we used were in accordance with the 2005 Chinese pharmacopoeia^20^. The details of the TXL capsule ingredient quality control standards and related research are in Supplementary Appendix III. Eligible subjects received TXL capsules, 6 pills twice daily, or placebo capsules, 6 pills twice daily for 24 months (see Supplementary Appendix VI Fig. S1). The study medications were provided by Shijiazhuang Yiling Pharmaceutical Co., China.

### Randomization and blinding

Randomization was performed in blocks of six (3: 3). An independent data centre named Interact Voice Responding System (IWRS) was used to complete the randomization according to a random sequence table (generated by SAS9.2). Pharmacists assigned numbers to the investigational product and placebo according to a random number table but did not participate in the trial. The details of randomization and blinding are in Supplementary Appendix IV.

### Ultrasonographic examination

All sonographers from participating medical centers received intensive training in the core laboratory for ultrasonography scanning of the carotid arteries according to the study protocol. Bilateral common and internal carotid arteries were scanned with an ultrasonography system equipped with a high frequency (7–11 MHz) probe at baseline and 12 and 24 months after enrollment. All information on research institutions, subjects and sonographers was digitally masked in images, and all images were sent to the core echo laboratory at Shandong University Qilu Hospital for analysis by two experienced physicians with commercially available software (TomTec Image-com) (see Supplementary Appendix V).

In the long-axis view, the IMT was measured along the anterior and posterior walls of the common and internal carotid arteries at 1 and 2 cm proximal to the carotid bifurcation in the common carotid artery and 1 cm distal to the carotid bifurcation in the internal carotid artery. Thus, a total of 12 sites in the bilateral carotid arteries were examined with the probe kept vertical to the surface of the skin (see Supplementary Appendix VI Fig. S2**)**.

In the carotid segment with the thickest IMT, both long- and short-axis views were obtained to detect plaque area, defined as a focal thickening of IMT ≥ 1.2 mm, which was ≥50% thicker than the IMT proximal or distal to the plaque. After a carotid plaque was detected, the maximal plaque area in the long-axis and short-axis view was traced manually (see Supplementary Appendix VI Figs S3 and S4).

For measuring the vascular remodeling index (RI), the carotid segments 5 mm proximal and distal to the maximal carotid plaque were chosen as the reference sites. The carotid artery diameter from the external elastic membrane of the anterior wall to that of the posterior wall was measured at the maximal plaque site and 2 reference sites, and the vascular RI was derived by calculating the ratio of the vessel diameter at the plaque site to the average of the vessel diameters at the reference sites. Vascular RI > 1.05 was considered positive remodeling, <0.95was considered negative remodeling, and 0.95–1.05 was considered no remodeling (see Supplementary Appendix VI Fig. S5**)**.

### Biochemical assay

The following biochemical parameters were measured at baseline and at 6, 12, 18 and 24 months: fasting serum levels of total cholesterol (TC), low-density lipoprotein cholesterol (LDL-C), high-density lipoprotein cholesterol (HDL-C), triglycerides (TG), high-sensitivity C-reactive protein (hs-CRP), glucose, glutamate pyruvate transaminase, glutamic-oxal (o) acetic transaminase, creatinine, blood urea nitrogen, serum creatine phosphokinase, prothrombin time and fibrinogen.

### Study outcomes

The primary outcome was between-group differences in the annualized change in mean IMT of 12 sites of bilateral carotid arteries over 24 months. The secondary outcomes were the change at 24 months in maximal plaque area; RI of the bilateral common carotid arteries; serum levels of lipids and high-sensitivity C-reactive protein (hs-CRP); and a composite outcome of major cardiovascular events (unstable angina pectoris, nonfatal myocardial infarction, cardiac death, revascularization, stroke and death) (see Supplementary Appendix VII). No interim analyses were planned or conducted.

### Statistical analysis

According to the results of the trial Measuring Effects on Intima-Media Thickness: an Evaluation of Rosuvastatin (METEOR)^21^, the annual increase in mean IMT at the 12 sites of the carotid artery is 0.012 mm (SD, 0.058) for patients with subclinical atherosclerosis. Assuming that the annual increase in mean IMT does not significantly differ in the TXL group, a minimum of 491 patients each in the TXL and placebo groups were required for 90% power to detect a between-group difference in change in mean carotid IMT of 0.012 mm (SD, 0.058) per year, at an alpha level of 0.05. Assuming a 20% dropout rate in each group, 590 patients in each group were needed. Outcome data were analyzed according to the intention-to-treat principle for all subjects who had undergone randomization. The primary outcome and secondary outcomes were analyzed with a multilevel repeated measures linear mixed-effects model specified in terms of fixed effects for group, time, and the interaction between group and time. The random effects within the model were intercept and slope for individual participants and sites within participants. All continuous values were expressed as mean ± SD except for changed outcome values from baseline to 12 or 24 months which were presented as least squares (LS) mean and 95% CI. Categorical data were expressed as percentages. The multiple Imputation and Pattern-Mixture model were used for sensitivity analysis of the primary outcomes^22^. The time to first major cardiovascular events were estimated by the Kaplan-Meier method, and the difference between the two groups was determined by the log-rank test. To test the reproducibility of ultrasonic measurements, four key variables, namely, IMT, plaque area in long- and short-axis views and RI, were re-measured in 50 randomly selected patients by two independent investigators. Inter-observer variability was assessed between the two investigators and intra-observer variability was assessed by one investigator at different times. Bland-Altman plots were used to analyze the inter- and intra-observer variability and intraclass correlation coefficients were calculated. The tests were two-tailed with nominal P values presented throughout without adjustment. All statistical analyses involved use of SAS 9.2 (SAS Inst., Inc., Cary, NC, USA).

## Results

**Figure 1:**
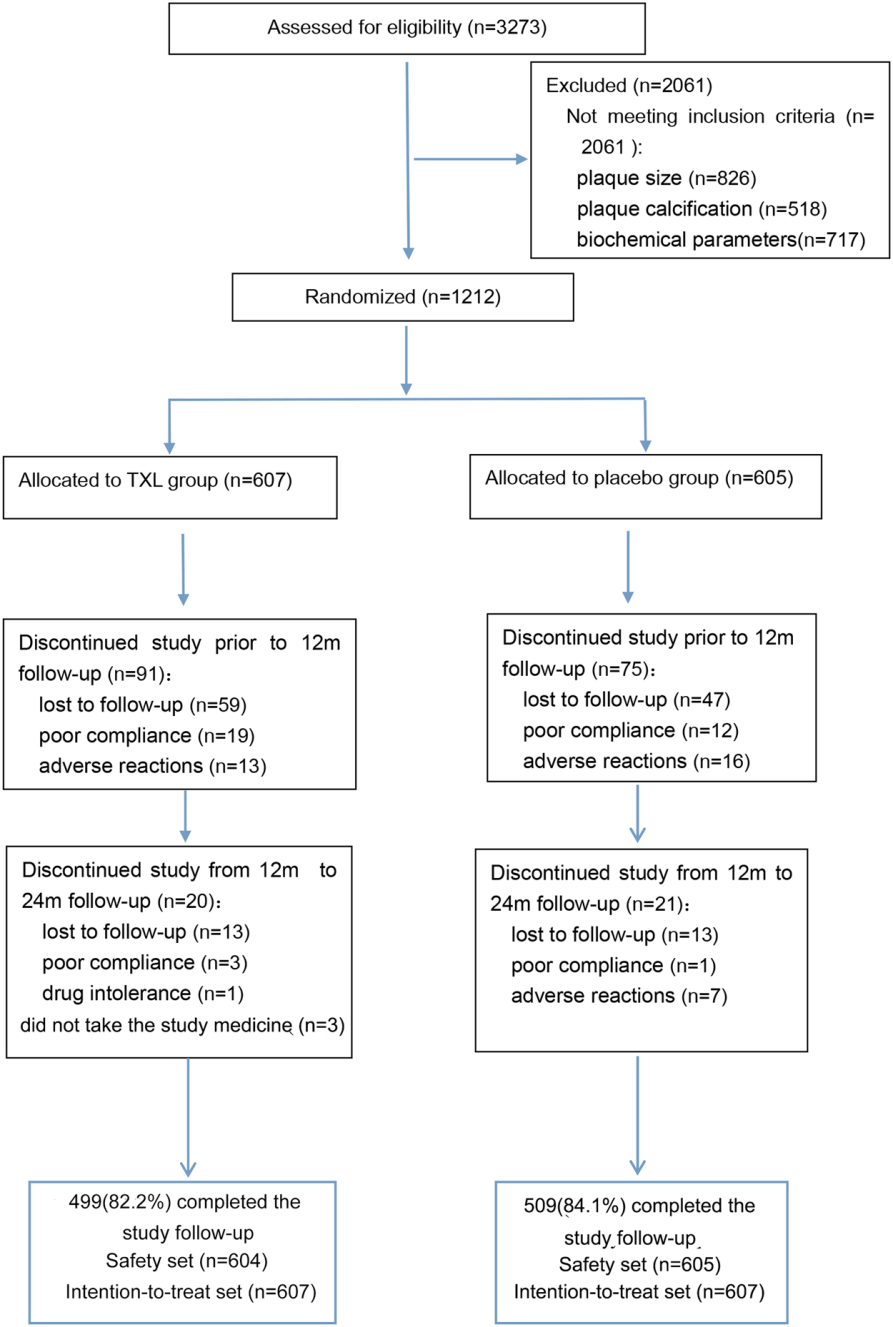
Trial Flow Diagram. A total of 1,212 eligible patients underwent laboratory assays and ultrasonography at baseline. Among them, 1008 participants (499 and 509 in the TXL and placebo group, respectively) completed the study follow-up.

We randomly assigned 1,212 eligible patients to the TXL group (n = 607) and placebo group (n = 605), all of whom underwent biochemical assessment and ultrasonography of bilateral carotid arteries at baseline. The first subject was enrolled in the trial in September 2009, recruitment ended in December 2011, and the follow-up end date was February 2014. During follow-up, the dropout rate was 17.8% (108) and 15.9% (96) in the TXL group and placebo group, respectively. Thus, 1008 participants (499 and 509 in the TXL and placebo group, respectively) completed the study follow-up (Fig. 1).

The demographic and clinical characteristics of the patients were in Table 1. The mean age of the TXL and placebo groups was 61.4 (SD, 8.4) and 61.4 (SD, 8.4) years, respectively, in the full analysis set, and 60.5% (367/607) and 58.7% (355/605) of the TXL and placebo groups, respectively, were men. Routine western medications were used in 75.1% of the patients, including statins for 36.4% and 33.9% in the TXL and placebo groups, respectively. Therefore, the two arms were balanced appropriately on baseline variables.

**Table 1:** Baseline Characteristics of Study Participants.

Characteristics	Tongxinluo (N = 607)	Placebo (N = 605)
Age(year)	61.4 ± 8.4	61.4 ± 8.2
Male sex—no. (%)	367 (60.5)	355 (58.7)
Body mass index(kg/m^2^)	24.7 ± 2.9	24.9 ± 2.9
Diabetes mellitus—no. (%)	128 (21.1)	113 (18.7)
Hypertension—no. (%)	314 (51.7)	342 (56.5)
Current smoker—no. (%)	162 (26.7)	174 (28.8)
History of myocardial infarction—no. (%)	25 (4.1)	18 (3.0)
Systolic blood pressure(mmHg)	129.4 ± 11.3	129.2 ± 11.4
Diastolic blood pressure(mmHg)	78.8 ± 7.8	79.0 ± 8.0
Serum glucose(mg/dl)	103.5 ± 20.7	102.4 ± 20.2
Medications at randomization—no. (%)	457 (75.3)	454 (75.0)
Aspirin	257 (42.3)	256 (42.3)
Statins	221 (36.4)	205 (33.9)
ACEI/ARB	156 (25.7)	185 (30.6)
Calcium antagonist	183 (30.1)	206 (34.0)

All data were mean ± SD or no. (%). The body-mass index is the weight in kilograms divided by the square of the height in meters. To convert values of serum glucose to millimoles per liter, multiply by 0.0555. ACEI/ARB, angiotensin-converting-enzyme inhibitor/angiotensin receptor blocker.

### Carotid intima-media thickness

**Figure 2:**
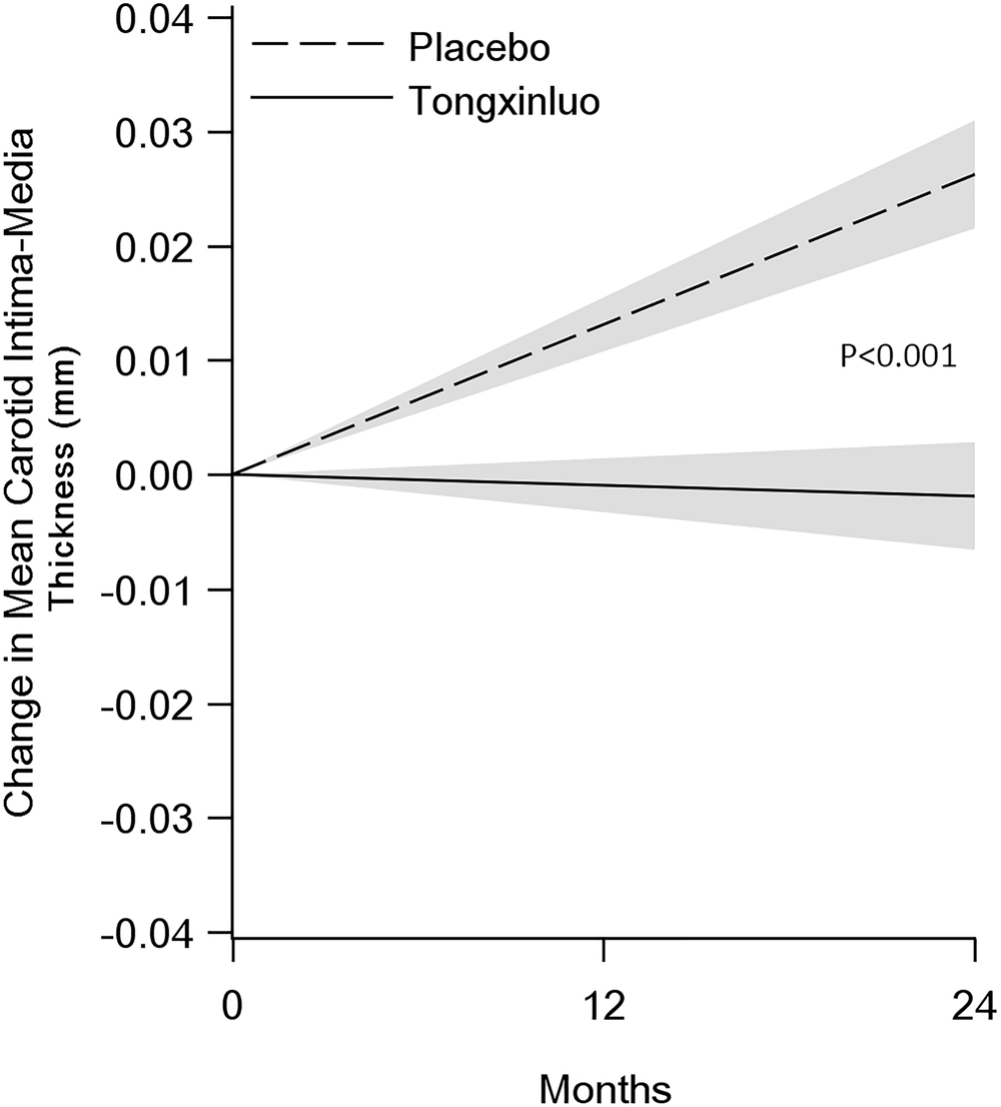
Effect of Tongxinluo or Placebo Treatment on Mean IMT. The annualized change in mean IMT of the TXL and placebo groups was −0.00095 mm (95% CI −0.00330~−0.00141) and 0.01312 mm (95% CI 0.01076~ 0.01548), respectively, and there was a significant difference between the two groups, with a group difference of −0.01407 (95% CI: −0.01740~−0.01073, p < 0.001).

At 24 months, the mean IMT in the TXL and placebo groups was 0.952 ± 0.151 mm and 0.970 ± 0.149 mm, respectively. Compared with the baseline measurements, the LS mean IMT was decreased by 0.0001 (95% CI: −0.0049~0.0048) in TXL group but increased by 0.0149 (95% CI: 0.0101~0.0197) in placebo group at 12 months, and the LS mean IMT was decreased by 0.0018 (95% CI: −0.0066~0.0031) in TXL group but increased by 0.0307 (95% CI: 0.0259~0.0355) in placebo group at 24 months (p for interaction <0.001, Table 2). The annualized change in mean IMT of the TXL and placebo groups was −0.00095 mm (95% CI: −0.00330~−0.00141) and 0.01312 mm (95% CI: 0.01076~0.01548), respectively, and there was a significant difference between the two groups, with a group difference of −0.01407 (95% CI: −0.01740~−0.01073, p < 0.001, Fig. 2).

**Table 2:** The Measured Values and Diachronical Analysis of Intima-media Thickness (IMT), Plaque Area and Remodeling Index (RI).

	Groups(n)	Baseline	12 months	24 months	*LS mean changes from baseline to 12 months	* LS mean changes from baseline to 24 months	#Interaction P values
**Mean IMT (mm)**							
	TXL(n = 607)	0.954 ± 0.156	0.953 ± 0.151	0.952 ± 0.151	−0.0001 (−0.0049,0.0048)	−0.0018 (−0.0066,0.0031)	<0.001
	Placebo (n = 605)	0.944 ± 0.148	0.957 ± 0.147	0.970 ± 0.149	0.0149 (0.0101,0.0197)	0.0307 (0.0259,0.0355)	
	Difference				−0.0150 (−0.0218, −0.0081)	−0.0325 (−0.0393, −0.0256)	
	P values				P < 0.001	P < 0.001	
**Plaque area in long-axis view (mm** ^2^ **)**							
	TXL (n = 607)	20.7 ± 14.3	20.0 ± 11.5	18.5 ± 9.3	0.105 (−0.678,0.889)	−0.513 (−1.399,0.373)	0.003
	Placebo (n = 605)	20.6 ± 14.3	20.4 ± 12.4	20.8 ± 12.0	0.790 (0.012,1.568)	1.671 (0.793,2.548)	
	Difference				−0.685 (−1.789,0.419)	−2.184 (−3.430, −0.937)	
	P values				P = 0.224	P < 0.001	
**Plaque area in short-axis view** (**mm**^2^**)**							
	TXL (n = 607)	19.9 ± 12.8	19.6 ± 11.5	19.3 ± 9.4	−0.217 (−1.145,0.711)	−0.234 (−1.289,0.821)	<0.001
	Placebo (n = 605)	19.7 ± 12.8	21.3 ± 13.3	22.6 ± 14.6	1.875 (0.960,2.791)	3.031 (1.995,4.066)	
	Difference				−2.093 (−3.396, −0.789)	−3.264 (−4.743, −1.786)	
	P values				P < 0.001	P < 0.001	
**Vascular remodeling index**							
RI < 0.95	TXL (n = 33)	0.918 ± 0.022	0.985 ± 0.082	1.103 ± 0.097	0.065 (0.028,0.102)	0.183 (0.145,0.222)	0.008
	Placebo (n = 30)	0.919 ± 0.026	1.005 ± 0.079	1.004 ± 0.110	0.085 (0.046,0.124)	0.086 (0.045,0.129)	
	Difference				−0.020 (−0.074,0.034)	0.098 (0.040,0.155)	
	P values				P = 0.468	P = 0.001	
0.95 ≤ RI ≤ 1.05	TXL (n = 261)	1.010 ± 0.02	1.041 ± 0.062	1.048 ± 0.085	0.032 (0.021,0.042)	0.038 (0.026,0.051)	0.210
	Placebo (n = 249)	1.012 ± 0.026	1.032 ± 0.075	1.061 ± 0.094	0.023 (0.012,0.033)	0.052 (0.040,0.065)	
	Difference				0.009 (−0.006,0.024)	−0.014 (−0.032,0.004)	
	P values				P = 0.249	P = 0.125	
RI > 1.05	TXL (n = 313)	1.120 ± 0.080	1.082 ± 0.085	1.061 ± 0.072	−0.047 (−0.061, −0.033)	−0.069 (−0.085, −0.054)	<0.001
	Placebo (n = 326)	1.125 ± 0.089	1.104 ± 0.125	1.104 ± 0.162	−0.026 (−0.039, −0.012)	−0.028 (−0.043, −0.013)	
	Difference				−0.022 (−0.041, −0.002)	−0.042 (−0.063, −0.020)	
	P values				P = 0.030	P < 0.001	

*Both primary and secondary outcomes were analyzed with linear mixed-effects model. All continuous values were mean ± SD except for changed outcome values from baseline to 12 or 24 months which were expressed as least squares (LS) mean and 95% CI. ^**#**^Interaction p value: the difference between the two groups induced by the interaction between treatment groups and time.

To rule out the possibility that the difference of the annualized change in mean IMT between the two groups might attribute to a slightly higher drop-out rate (17.8%) in TXL group than in the placebo group (15.9%), we performed sensitivity analysis of the primary outcomes with two methods: (1) Assuming data were missing at random, the multiple imputation method was used and the derived mean difference of the annualized change in mean IMT between the two groups was −0.01693 (95% CI, −0.02155 to −0.01230, p < 0.001); (2) Assuming data were missing not at random, the Pattern-Mixture model was used and the calculated mean difference of the annualized change in mean IMT between the two groups was −0.01663 (95% CI, −0.02126 to −0.01200, p < 0.001). Thus, the results from the two sensitivity analyses were consistent with the primary outcome analysis, i.e., there was no evidence of a bias due to the differential missing data between the groups.

### Carotid plaque area

**Figure 3:**
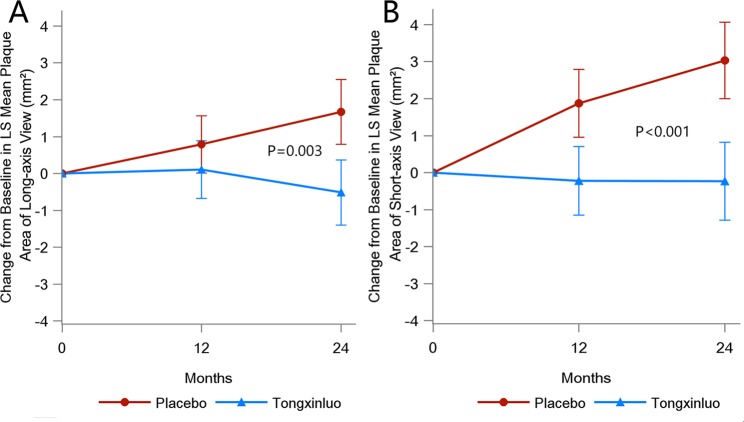
Change from Baseline in LS Mean Plaque Area in the Long-axis (**A**) and Short-axis (**B**) Views. The p values compared the difference in LS mean changes of plaque area between the TXL and placebo groups over 24 months. The vertical bars indicated 95% confidence interval (CI).

Relative to the baseline measurements, the LS mean of the maximal plaque area in the long-axis view of the bilateral carotid arteries decreased by 0.513 (95% CI: −1.399~0.373) in the TXL group but increased by 1.671 (95% CI: 0.793~2.548) in the placebo group at 24 months (Table 2). There was a significant difference in the change in plaque area from baseline to 24 months in the long-axis view between the two groups (P = 0.003, Fig. 3). Likewise, in comparison with the baseline values, the LS mean of the maximal plaque area in the short-axis view of the bilateral carotid arteries decreased by 0.234 (95% CI: −1.289~0.821) in the TXL group but increased by 3.031 (95% CI: 1.995~4.066) in the placebo group at 24 months (Table 2). There was also a significant difference in the change of plaque area from baseline to 24 months in the short-axis view between the two groups (P < 0.001, Fig. 3).

### Vascular remodeling index

All patients were divided into three subgroups by their carotid artery RI values measured at baseline: <0.95, 0.95–1.05 and >1.05. In patients with RI < 0.95, RI was increased in TXL group only at 24 months relative to the baseline values (p = 0.001, p for interaction = 0.008). In patients with 0.95 ≤ RI ≤ 1.05, the two groups did not differ in changes of RI over time (p = 0.125, p for interaction = 0.210).In patients with RI > 1.05, RI was decreased in both groups at 12 and 24 months compared with baseline values, and the LS mean of changes from baseline to 24 months was greater in the TXL than the placebo group (p < 0.001, p for interaction < 0.001) (Table 2).

### Biochemical measurements

The LS mean changes from baseline to 24 months of serum levels of LDL-C, TC and TG were increased in the placebo group but decreased in the TXL group with a significant difference between the two groups (p = 0.029, p < 0.001 and p = 0.028, respectively), whereas the serum levels of HDL-C levels showed opposite changes in the TXL and placebo groups with a significant between-group difference (P = 0.003). However, the p values for interaction were all >0.05 for serum levels of LDL-C, TC and TG, indicating that there was no evidence of a difference between the two groups in serum lipid changes over time. In addition, there was no significant difference between the two groups in LS mean changes from baseline to 24 months of serum hs-CRP levels (P = 0.127) (Table 3).

**Table 3:** Biochemical Measurements at Baseline and during Follow-Up.

	Baseline	12 months	24 months	LS mean change from baseline to 12 months	LS mean change from baseline to 24 months	#Interaction P values
**LDL-C (mg/dl)**						
Tongxinluo (N)	113.63 ± 32.96 (595)	115.05 ± 32.29 (487)	113.75 ± 31.23 (470)	0.659 (−1.921, 3.240)	−1.007 (−3.621, 1.607)	0.283
Placebo (N)	113.23 ± 32.23 (597)	116.16 ± 34.77 (492)	116.62 ± 33.86 (484)	1.911 (−0.669,4.492)	3.0781 (0.506, 5.651)	
Difference				−1.252 (−4.902, 2.398)	−4.085 (−7.754, −0.417)	
P values				0.501	0.029	
**TC (mg/dl)**						
Tongxinluo (N)	189.05 ± 43.11 (596)	190.82 ± 43.05 (488)	188.15 ± 38.52 (472)	1.475 (−1.743, 4.694)	−1.634 (−4.891, 1.622)	0.264
Placebo (N)	186.22 ± 39.75 (597)	193.25 ± 43.06 (496)	193.27 ± 41.01 (486)	5.701 (2.487, 8.912)	6.258 (3.053, 9.463)	
Difference				−4.225 (−8.775, 0.325)	−7.892 (−12.465, −3.319)	
P values				0.069	<0.001	
**TG (mg/dl)**						
Tongxinluo (N)	140.82 ± 78.72 (596)	143.56 ± 75.56 (489)	142.85 ± 78.09 (473)	1.899 (−2.787, 6.584)	−0.148 (−4.889, 4.593)	0.943
Placebo (N)	137.41 ± 81.69 (597)	146.27 ± 83.83 (496)	144.70 ± 85.74 (485)	9.014 (4.332, 13.696)	7.310 (2.634, 11.985)	
Difference				−7.116 (−13.741, −0.490)	−7.458 (−14.118, −0.798)	
P values				0.035	0.028	
**HDL-C (mg/dl)**						
Tongxinluo (N)	50.43 ± 13.75 (596)	51.17 ± 12.35 (486)	51.56 ± 12.40 (470)	0.644 (−0.295, 1.583)	1.273 (0.323, 2.223)	0.346
Placebo (N)	51.16 ± 12.89 (597)	50.58 ± 12.79 (492)	49.92 ± 13.45 (484)	−0.459 (−1.397, 0.480)	−0.734 (−1.669, 0.202)	
Difference				1.102 (−0.226, 2.430)	2.007 (0.673, 3.340)	
P values				0.104	0.003	
**hs-CRP (mg/l)**						
Tongxinluo (N)	2.03 ± 2.06 (458)	1.92 ± 2.35 (350)	2.04 ± 1.97 (337)	−0.095 (−0.317, 0.128)	−0.011 (−0.237, 0.215)	0.636
Placebo (N)	1.87 ± 2.03 (473)	1.97 ± 2.42 (371)	2.18 ± 2.42 (344)	0.045 (−0.172, 0.263)	0.236 (0.013, 0.459)	
Difference				−0.140 (−0.452, 0.171)	−0.247 (−0.565, 0.070)	
P values				0.378	0.127	

All continuous values were mean ± SD except fort changed outcome values from baseline to 12 or 24 months which were analyzed by linear mixed-effects model and expressed as least squares (LS) mean and 95% CI. **#**Interaction p value: the difference between the two groups induced by the interaction between treatment groups and time. LDL-C: low-density lipoprotein cholesterol; TC: total cholesterol; TG: triglycerides; HDL-C: high-density lipoprotein cholesterol; hs-CRP: high-sensitivity C-reactive protein. To convert values of LDL-C, TC and HDL-C to millimoles per liter, multiply by 0.0259. To convert values of TG to millimoles per liter, multiply by 0.0113.

### Major cardiovascular events

**Figure 4:**
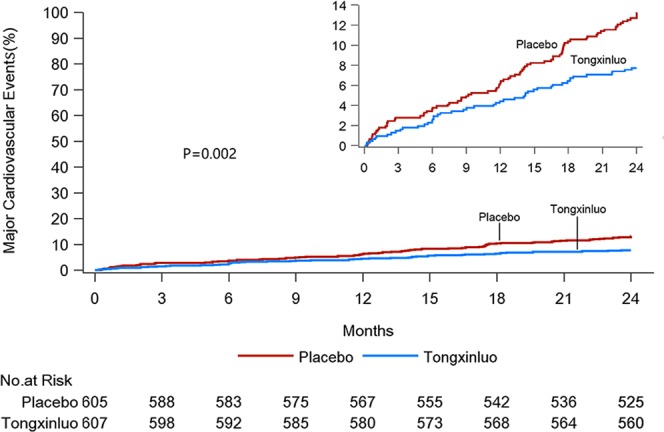
Kaplan-Meier Estimates of the Effect of Tongxinluo or Placebo Treatment on Incidence of Major Cardiovascular Events. Curves indicate the first event rates of unstable angina pectoris, nonfatal myocardial infarction, cardiac death, revascularization, stroke and death in the intent-to-treat population from randomization to the first occurrence of a cardiovascular event, the last office or phone visit, or the day of death during follow-up. The inset upper right corner shows the same data on an enlarged axis.

From Kaplan-Meier estimates, time to first major cardiovascular events was later and the event rate was lower, in the TXL group than in the placebo group. Cardiovascular events occurred in 47 patients (7.7%) of the TXL group and in 80 patients (13.2%) of the placebo group, (P = 0.002), and in particular, the occurrence of unstable angina pectoris was lower in the TXL than placebo group (P = 0.005) (Fig. 4, Supplementary Appendix IX: Table S1).

### Drug-related adverse events

The TXL and placebo groups did not differ in the adverse reactions reported. Adverse events considered related to treatment occurred in 55/604 patients (9.1%) and 45/605 patients (7.4%) in the TXL and placebo groups, respectively (P = 0.29), with epigastric discomfort and headache being the most common complains. The compliance analysis based on capsule counting showed that patient adherence to taking study medications was 98.2% and 99.8% in the TXL and placebo groups, respectively. Discontinuation of study medications owing to an adverse event occurred in 2.2% (13/604) and 3.8% (23/605) of patients in the TXL and placebo groups, respectively **(see** Supplementary Appendix IX: Table S2**)**.

### Ultrasonographic measurement reproducibility

**Figure 5:**
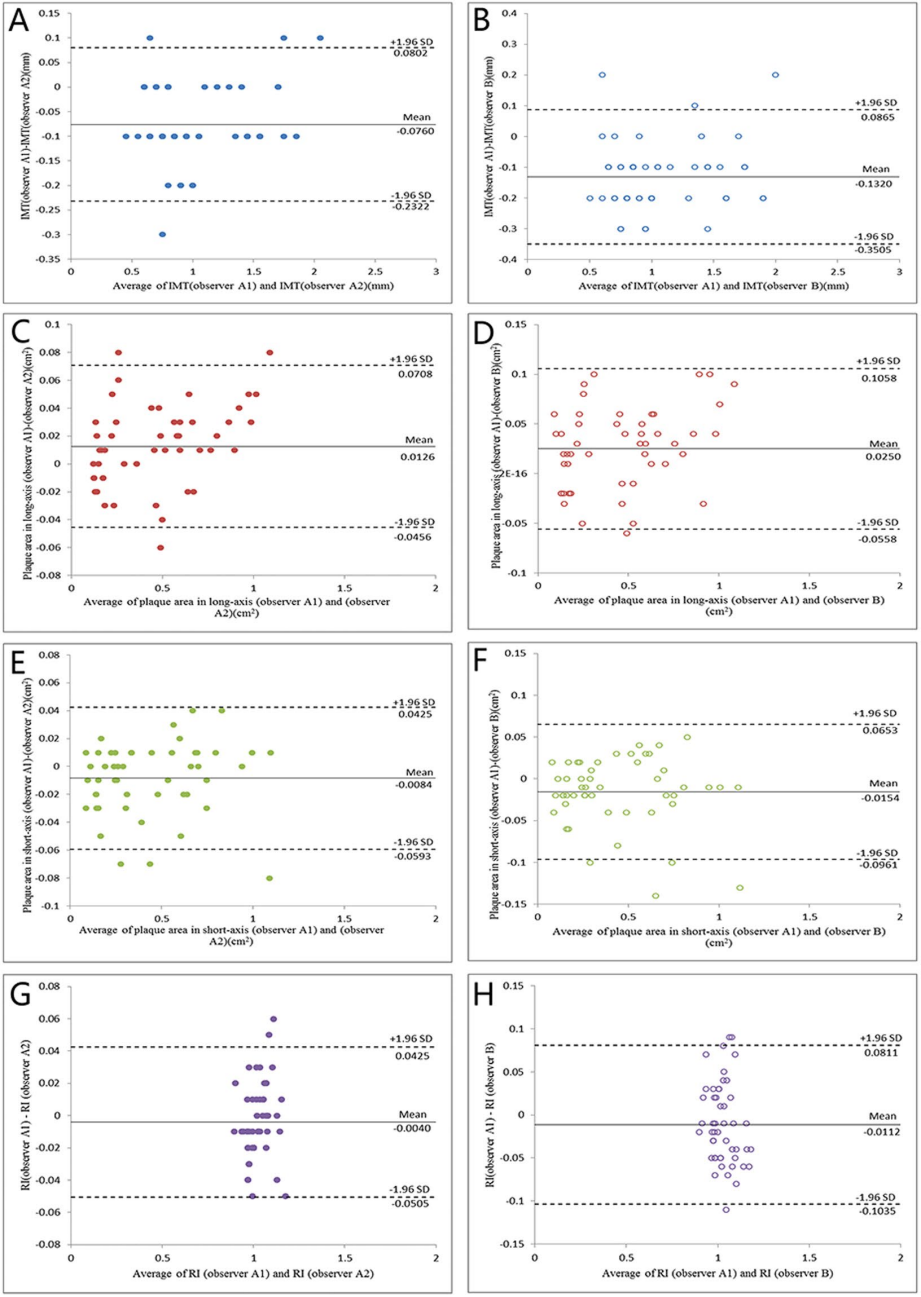
Bland-Altman Plots of Intra-observer and Inter-observer Variability in Measurement of IMT (**A**,**B**), Plaque Area in long-axis views (**C**,**D**) and short-axis views (**E**,**F**) and Vascular Remodeling Index (**G**,**H**). The intraclass correlation coefficients of intra-observer variability for IMT, plaque area in long- and short-axis views and RI were 0.982, 0.915, 0.906 and 0.905, respectively (P < 0.001 for all), and those of inter-observer variability for IMT, plaque area in long- and short-axis views and RI were 0.963, 0.911, 0.890 and 0.804, respectively (P < 0.001 for all).

The intraclass correlation coefficients of intra-observer variability for IMT, plaque area in long- and short-axis views and RI were 0.982, 0.915, 0.906 and 0.905, respectively (P < 0.001 for all), and those of inter-observer variability for IMT, plaque area in long- and short-axis views and RI were 0.963, 0.911, 0.890 and 0.804, respectively (P < 0.001 for all) (Fig. 5).

## Discussion

In the present CAPITAL study, we showed that in a large cohort of Chinese patients with carotid atherosclerotic plaque and at high risk for cardiovascular events, in addition to current routine therapy, administration of TXL capsules for 24 months retarded the progression of the mean IMT and maximal plaque area of the carotid artery. In addition, TXL treatment significantly attenuated positive vascular remodeling of the carotid artery and reduced the incidence of unstable angina. Recently, the progression of atherosclerotic plaques as an important predictor of cardiovascular events has received increasing attention. Several imaging studies have demonstrated rapid lesion progression and consequent luminal obstruction before the onset of acute coronary syndrome in most patients^23,24^. These lesions often have larger plaque volume and a necrotic core with greater positive vessel remodeling compared with silent plaques. The possible mechanisms underlying rapid plaque progression may involve recurrent plaque rupture and healing and intraplaque neovascularization and hemorrhage with deposition of erythrocyte-derived free cholesterol^25^. In addition, intravascular ultrasound studies showed that patients resistant to statin therapy often exhibit rapid atheroma progression^26^. Therefore, measurement of the rate of plaque progression might provide a new approach to identifying vulnerable plaques with increased potential for adverse outcomes.

In the present study, we used two ultrasound variables, mean IMT and maximal plaque area, to measure the rate of plaque progression in the carotid artery. Early clinical trials compared lovastatin or rosuvastatin to placebo for their efficacy on the progression of carotid mean IMT and found that both statins had a superior effect^14,15^. However, when combined with simvastatin, ezetimibe was not superior to placebo at affecting progression of carotid mean IMT in patients with familial hypercholesterolemia^16^. In the present study, we compared TXL to placebo in terms of efficacy on the progression of carotid mean IMT in addition to routine therapies including aspirin, statins, ACEI/ARBs and calcium antagonists, and found that TXL had a positive effect. The Asymptomatic Carotid Artery Progression Study (ACAPS) found that a carotid IMT reduction of 0.009 mm/year was associated with a significant reduction in major cardiovascular events^14^. In the METEOR trial, the progression of carotid mean IMT was −0.0014 and 0.0131 mm/year with rosuvastatin and placebo treatment, respectively^15^. Our study showed carotid mean IMT progression of −0.00095 and 0.01312 mm/year for the TXL and placebo groups, respectively, and the occurrence of unstable angina was lower in the TXL than the placebo group at 24 months. These results were better than those of the ACAPS and METEOR trials because in the latter two trials, no anti-atherosclerotic medications were given in the placebo groups.

Recent studies reported that carotid plaque area was associated more closely than mean IMT with cardiovascular events^18,19^, probably because carotid plaque may reflect cumulative damage from exposure to different atherosclerotic risk factors and thus is more specific than IMT at reflecting atherosclerotic burden. In the present study, we found that maximal plaque area measured from both long- and short-axis views over 24 months significantly increased in the placebo group but remained unchanged in the TXL group, leading to a substantial difference in the maximal plaque area between the two groups. These results agreed with the carotid mean IMT measurements and verified the therapeutic effects of TXL in retarding the progression of atherosclerotic lesions.

Vascular remodeling refers to compensatory vascular expansion (positive remodeling) or constriction (negative remodeling) in response to plaque growth. Our previous study demonstrated that in patients with angina pectoris, coronary RI was an independent predictor of coronary plaque rupture^27^. A recent meta-analysis confirmed that RI measured by CT angiography was an independent predictor of future acute coronary syndrome^28^. In the present study, RI was significantly increased in patients with RI < 0.95 at baseline but substantially decreased after TXL treatment in those with RI > 1.05 at baseline. These results agreed with the carotid plaque measurements and suggest that TXL treatment might have potential to stabilize atherosclerotic plaques in high-risk patients. The significant reduction in major cardiovascular events, notably the incidence of unstable angina, in patients receiving TXL in our study further corroborated the anti-atherosclerotic effects of TXL treatment.

The mechanisms underlying the anti-atherosclerotic effects of TXL treatment are not completely understood. The absolute difference in serum lipid levels between the two groups at the end of the study was too small to explain the morphological changes in carotid plaque probably because the mean serum lipid levels in the two groups were within the normal limits according to the latest Chinese guidelines^29^. These results were in contrast with previously reported studies of anti-atherosclerotic therapies showing greatly elevated serum lipid levels in patients and animals at baseline^4,5,15,16^. Another possible mechanism is a TXL-mediated anti-inflammatory effect. TXL treatment may reduce the incidence of recurrent plaque rupture and healing, thus retarding the progression of carotid plaque, which is supported by the TXL treatment lowering the incidence of unstable angina pectoris in our patients. A more recent study in our laboratory revealed that TXL treatment dose-dependently reduced the vasa vasorum proliferation of atherosclerotic plaques by inhibiting the local expression of inflammatory cytokines and angiogenesis factors in ApoE−/− mice^5^, which may prevent intraplaque neovascularization and hemorrhage, thereby restraining plaque growth.

The drug-related adverse events occurred in 9.1% patients and 7.4% patients in the TXL and placebo groups, respectively. However, discontinuation of study medications owing to an adverse event occurred in 2.2% and 3.8% of patients in the TXL and placebo groups, respectively. These results indicate that TXL is a safe medication and the slightly higher drop-out rate in the TXL than in the placebo group cannot be explained by the side effects induced by TXL treatment.

Several limitations of the study need to be addressed. First, measurement of the carotid mean IMT and plaque area over time is subject to errors that may cause variations in derived values. All sonographers received intensive training in the core laboratory to ensure sufficient technical skill, and all images were analyzed in the core laboratory. The high inter- and intra-observer reproducibility obtained in this study corroborated the reliability of ultrasonographic measurements. Second, the TXL capsule is a mixture of 12 plant and animal products, and the effective anti-atherosclerotic component in this medication is unclear. However, isolation of the effective molecule(s) from TXL capsules is an extremely difficult task, even when using current pharmaceutical technology, and we believe validation of the efficacy and safety of TXL capsules is the first step forward. Third, the drop-out rate during follow-up seemed slightly higher in the TXL group (17.8%) than in the placebo group (15.9%), which might affect the outcome difference between the two groups. However, our sensitivity analysis demonstrated that the difference in the primary outcome between the two groups remained significant no matter what statistical method was used.

In conclusion, our CAPITAL study demonstrates that in patients with subclinical carotid atherosclerosis, in addition to routine therapy, TXL treatment retarded the progression of mean IMT, plaque area and vascular remodeling of the carotid artery, with a good safety profile. Further randomized clinical trials are warranted to confirm the therapeutic effects of TXL treatment on the long-term outcome of patients at high risk for cardiovascular events.

## Supplementary information

supplemental material

## Data Availability

All the data of this manuscript is available.
