# Supplementary material for: Carotid artery plaque intervention with Tongxinluo capsule (CAPITAL): A multicenter randomized double-blind parallel-group placebo-controlled study
Source: Sci Rep. 2019 Mar 14;9:4545. doi: 10.1038/s41598-019-41118-z (PMC6418108; doi:10.1038/s41598-019-41118-z)
Supplement: Supplementary file 1 — supplemental material [file 41598_2019_41118_MOESM1_ESM.docx]

**Supplementary**

**Carotid artery plaque intervention with Tongxinluo capsule (CAPITAL): A multicenter randomized double-blind parallel-group placebo-controlled study**

Mei Zhang, MD^1#^; Yan Liu, MD^1#^; Mingjun Xu, MD^1^; Lei Zhang, MD^1^; Yan Liu, MD^1^; Xiaoling Liu, MD^1^; Yuxia Zhao, MD^1^; Fang Zhu, MD^2^; Rui Xu, MD^3^;Zhihong Ou, MD^4^; Ying Wang, MD^5^; Qigong Liu, MD^6^; Shuping Ma, MD^7^; Tian Wang, MD^8^; Maolin He, MD^9^; Qinghua Lu, MD^10^; Honghua Li, MD^11^; Jihan Huang, PhD^12^; Yun Zhang, MD*^1^.

From the ^1^Department of Cardiology, Qilu Hospital of Shandong University, Jinan, China; ^2^Department of Cardiology, People's Hospital of Liaoning Province, Shen Yang, China; ^3^ Department of Cardiology, Shandong Provincial Qianfoshan Hospital, Jinan, China; ^4^ Department of Cardiology, People's Hospital of Linyi City, Lin Yi, China; ^5^ Department of Cardiology, Second Affiliated Hospital of Shandong Traditional Chinese Medicine University, Jinan, China; ^6^ Department of Cardiology, Hospital Affiliated with Tongji Medical College of Huazhong University of Science and Technology, Wuhan, China; ^7^ Department of Cardiology, Hebei Provincial People's Hospital, Shi Jiazhuang, China; ^8^ Department of Cardiology, Traffic Hospital of Shandong Province, Jinan, China; ^9^ Department of Cardiology, Beijing Shijitan Hospital, Beijing, China; ^10^ Department of Cardiology, Second Affiliated Hospital of Shandong University, Jinan, China; ^11^ Department of Cardiology, Wuhan General Hospital of Guangzhou Military Region, Wuhan (H.H.L.); ^12^ Center for Drug Clinical Research, Shanghai University of Traditional Chinese Medicine, Shanghai, China.

#These two authors contributed equally.

**Address for correspondence:** *Prof. Yun Zhang, MD, FACC, FESC, FASE, The Key Laboratory of Cardiovascular Remodeling and Function Research, The Key Laboratory of Cardiovascular Remodeling and Function Research, Chinese Ministry of Education, Chinese National Health Commission and Chinese Academy of Medical Sciences, The State and Shandong Province Joint Key Laboratory of Translational Cardiovascular Medicine, Department of Cardiology, Qilu Hospital of Shandong University, No. 107, Wenhuaxi Road, Jinan, 250012, PR. China. Email: [zhangyun@sdu.edu.cn](mailto:zhangyun@sdu.edu.cn). [TEL: +86531-82169257](TEL:+86531-82169257), FAX: +86531-86169356,

Email: [zhangyun@sdu.edu.cn](mailto:zhangyun@sdu.edu.cn),[TEL: +86531-82169257](TEL:+86531-82169257), FAX: +86531-86169356.

**Table of contents**

Supplementary appendix I: List of committee and board members and other investigators for CAPITAL trial Page 4

Supplementary appendix II: Criteria for subject withdraw and study termination

Page 12

Supplementary appendix III: Quality control standard of Tongxinluo capsule ingredients and related research Page 13

Supplementary appendix IV: The randomization and blinding Page 28

Supplementary appendix V: Instructions for carotid artery ultrasonography examination and image analyses Page 30

Supplementary appendix VI: Supplementary figures Page 34

Supplementary appendix VII: Definition of major cardiovascular events Page 39

Supplementary appendix VIII: Research evidence in context Page 41

Supplementary appendix IX: Supplementary tables Page 44

**Supplementary appendix I: List of committee and board members and other investigators for CAPITAL trial**

**Committee and board members**

**Consultative Committee:**

Chair:

Boli Zhang, MD

China Academy of Traditional Chinese Medicine

E-mail: [zhangbolipr@163.com](mailto:zhangbolipr@163.com)

Tel: 8613502063466

**Board Members:**

Runlin Gao, MD

Fuwai Hospital of Cardiovascular Disease Chinese Academy of Medical Sciences

E-mail: [gaorunlin@citmd.com](mailto:gaorunlin@citmd.com)

Fax: 861068331622

Jingxuan Guo, MD

The Third Hospital of Peking University

E-mail: [jianliu68@gmail.com](mailto:jianliu68@gmail.com)

Tel: 8613910688497

**Executive Steering Committee:**

Chair:

Yun Zhang, MD

Department of Cardiology, Key Laboratory of Cardiovascular Remodeling and Function Research, Chinese Ministry of Education and Chinese Ministry of Health, Qilu Hospital of Shandong University

Email: [zhangyun@sdu.edu.cn](mailto:zhangyun@sdu.edu.cn)

Fax: 8653186169356

**Board Members:**

Yuejin Yang, MD

Fuwai Hospital of Cardiovascular Disease Chinese Academy of Medical Sciences

E-mail: [yangyjfw@126.com](mailto:yangyjfw@126.com)

Tel: 8613701151408

Jianan Wang, MD

The Second Affiliated Hospital of Medical College of Zhejiang University

E-mail：[wja@zju.edu.cn](mailto:wja@zju.edu.cn)

Tel: 8613805786328

Xinchun Yang, MD

Beijing Chaoyang Hospital affiliated to Capital Medical University

E-mail: [yxc6229@sina.com](mailto:yxc6229@sina.com)

Tel: 8613701186229

Bo Yu, MD

The Second Affiliated Hospital of Harbin Medical University

E-mail：[yubodr@163.com](mailto:yubodr@163.com)

Tel: 8613804585601

**Clinical Coordinating Center:**

Chair:

Mei Zhang, MD

Qilu Hospital of Shandong University

E-mail: [daixh@vip.sina.com](mailto:daixh@vip.sina.com)

Tel: 8618560086629

**Board Members:**

Rui Xu, MD

Shandong Provincial Qianfoshan Hospital

E-mail: [xuruicn@hotmail.com](mailto:xuruicn@hotmail.com)

Tel: 8615965311789

Tian Wang, MD

Shandong Traffic Hospital

E-mail: [wt1118@163.com](mailto:WT1118@163.com)

Tel: 8613964165892

**Data and Safety Monitoring Board:**

Chair:

Dingyin Zeng, MD

The First Affiliated Hospital of China Medical University

E-mail:zenghetao@hotmail.com

Tel: 8613889100055

**Board Members:**

Yundai Chen, MD

Chinese PLA General Hospital

E-mail:cyundai@medmail.com.cn

Tel: 8613311119778

Bo Cao, MD

Public Education Department of Peking University Health Science Center

E-mail: [74050@bjmu.edu.cn](mailto:74050@bjmu.edu.cn)

Fax: 861082805362

**Clinical end Point Committee:**

Chair:

Zonggui Wu, MD

Changzheng Hospital of the Second Military Medical University

E-mail:zgwu@medmail.com.cn

Tel: 8613511828263

**Board Members:**

Weikang Wu, MD

Institute of Combined Traditional Chinese and Western Medicine of Zhongshan University

E-mail: [w2004wk@aliyun.com](mailto:w2004wk@aliyun.com)

Tel: 8613711498781

Shijie You, MD

Fuwai Hospital of Cardiovascular Disease Chinese Academy of Medical Sciences

E-mail: [youshijie6001@aliyun.com](mailto:youshijie6001@aliyun.com)

Tel: 8613601200351

**Study Statistician:**

Jihan Huang, PhD

Center for Drug Clinical Research, Shanghai University of Traditional  Chinese Medicine

 E-mail: [huangjihan@21cn.com](mailto:huangjihan@21cn.com)

Tel: 8615000308719

**Data Management:**

Yan Liu, MD

Qilu Hospital of Shandong University

E-mail: liuyan200751@126.com,

Tel: 8618678817863

Mingjun Xu, MD

Qilu Hospital of Shandong University

E-mail: xmj223@163.com

Tel: 8618560086569

**Central Laboratory:**

Qilu Hospital of Shandong University, 107 Wenhuaxi Road, Jinan, China.

**Other investigators for CAPITAL trial**

Qilu Hospital of Shandong University, Guihua Yao, Mei Ni, Fangfang Liu, Xiaowei Wang, Lekang Zhao, Xiao Wu，Pengfei Zhang, Ming Zhong, Peili Pu,;

Beijing Chaoyang Hospital, Beijing, Xinchun Yang, Liping Yu, Yafeng Wu, Wei Jiang;

Qingdao Municipal Hospital, Qingdao, Weiqiang Kang, Xin Wang, Wei Kong, Yansong Li;

The Second Hospital of Tianjin Medical Hospital, Tianjin, Lifeng Li, Huaying Fu;

Jiangxi Pingxiang People’s Hospital, Nanchang, Fei Yi, Qiong He, Xihui Hu, Yongzhong Yi;

Sichuan Provincial People’s Hospital, Chengdu, Xiaofang Zhou, Wenyan Wang, Chunmei Li;

The Central Hospital of Jinan City, Jinan, Guohai Su, Xiaojun Cai, Zhenhua Li;

Nanchang University Forth Affiliated Hospital, Nanchang, Xiaoliang Lou, Xiaoping Li, Hua Fan, Jianping Tan;

The First Affiliated Hospital of Nanjing Medical University, Nanjing, Kejiang Cao, Liansheng Wang, Jun, Zhu;

Chengdu Second People’s Hospital, Chengdu, Yongmei Hu, Xiaoqiang Zhang, Yan Wang;

Yantai Yuhuangding Hospital, Yantai, Jun Yang, Xiaotong Wang, Li Wang, Yan Pan;

The second Affiliated Hospital Haerbin Medical University, Haerbin, Bo Yu, Jiawei Tian, Yibo Guo, Shuangquan Jiang;

The Second Hospital of Lanzhou University, Lanzhou, Jing Yu, Xin Lin, Hongxia Lu;

The Second Affiliated Hospital of Zhejiang University School, Zhejing, Jianan Wang, Xiaojie Xie;

The Sixth Hospital of Shanghai City, Shanghai, Xiaojiang Sun, Yan Wang;

Shanxi Provincial Peopole’s Hospital, Xian, Yunzhang Song, Hua Lv, Hong Zhang, Yamei Shen;

The Second Facilitated Hospital of Fujian Medical University, Fuzhou, Ying Ye;

Shandong Provincial Traditional Chinese Medical Hospital, Jinan, Feng Lu, Zhen Wang, Lei Li;

The First Affiliated Hospital of Anhui Medical University, Hefei, Zhongwu Sun, Ling Wang, Hui Zheng;

The First Affiliated Hospital of Guangxi Medical University, Nanning, Jinou Zheng;

Handan Central Hospital, Handan, Juntao Li, Huiyong Huo;

Taian Central Hospital, Taian, Yun Wu;

The Affiliated Hospital of Medical College Qingdao University, Qingdao, Shanglang Cai, Yang Ji, Pin Sun;

Yantaishan Hospital, Yantai, Juexin Fan, Zhiying Xu, Shuyan Jiang;

Fuwai Hospital of Cardiovascular Disease, Bejing, Yuejin Yang, Jinhui Song, Yong Jiang, Jianpeng Wang;

The People's Hospital of Hebei Province, Shijiazhuang, Hongyuan Xue, Li Gao;

Tongji Hospital Affiliated to Tongji Medical College Huazhong University of Science & Technology, Wuhan, Daowen Wang, Haoyi Yang, Wei Zhang, Na Chen;

Qianfoshan Hospital of Shandong Province, Jinan, Li Zhang, Qing Wang, Xiaojun Wang, Mei Gao;

The People's Hospital of Liaoning Province, Shenyang, Hanzhang Zhao, Mingyan Ding, Tong Li;

The Second Hospital of Shandong University, Jinan, Jingbo Zhang, Fengtao Wei, Yongmei Wang;

Shandong Jiaotong Hospital, Jinan, Haiyan Meng, Qizhi Zhang, Wancai Cao;

The Second Affiliated Hospital of Shandong University of Traditional Chinese Medicine, Jinan, Fang Liu, Jun Chen;

Beijing Shijitan Hospital, Beijing, Wenhong Liu, Feng Yu;

Linyi People's Hospital, Linyi, Guiling Sun, Xue Wang;

Wuhan General Hospital of Guangzhou Military, Wuhan, Pei Zhou, Zhipeng Xu, Wenjing Luo.

**Supplementary appendix II:  Criteria for subject withdraw and study termination**

**Criteria for subject withdraw**

(1) Drug-related anaphylaxis;

(2) Drug-related abnormal hemorrhage, symptoms, signs and laboratory test results that investigators believe to be severe enough to withdraw subjects from the study;

(3) Pregnancy during the study;

(4) Subject’s request to withdraw;

(5) Poor tolerance to treatment;

(6) Lost to follow-up;

(7) Poor compliance;

(8) Emergent unblinding or unexpected data leak

(9) Drug-induced adverse events

**Criteria for study termination**

(1) Investigator-reported serious safety issues;

(2) Poor effects making continued study unnecessary;

(3) Serious mistakes in study design;

(4) Lack of sufficient research funding;

(5) Request from the administrative authorities to terminate the study.

Study termination may be temporary or permanent. All study records should be kept for inspection after study termination.

**Supplementary appendix III: Quality control standard of Tongxinluo capsule ingredients and related research**

Tongxinluo capsule consists of Ginseng radix et rhizoma, Hirudo, Scorpio, Paeoniae rubra radix, Periostracum cicadae, Eupolyphaga seu steleophaga, Scolopendra, Lignum santali alba, Lignum dalbergiae odoriferae, Olibanum (processed), Ziziphi spinosae, Semen (stir-baked), and Borneolum syntheticum.

**Descriptions**

Tongxinluo capsule is a hard capsule containing pale brown to brown granules and powder, with an aromatic odour and slightly salty and bitter taste.

**Identification**

Put 5 g of the contents of Tongxinluo capsules into 60 ml of water and the solution is filtered or centrifuged after ultrasonic treatment for 20 min. Evaporate the filtrate (or the supernatant) to dryness and dissolve the residue with 20 ml methanol before filtration. Apply the filtrate to a column (1 cm in diameter) packed with neutral alumina (100-200 mesh, 3 g), elute it with l0 ml methanol and discard the methanol eluate. Elute the column again with 30 ml water, collect the water eluate and evaporate it to dryness. Dissolve the residue with l ml methanol to produce a test solution. Prepare a reference drug solution by adding 30 ml water to 1 g Hirudo as a reference drug and processing in the same manner. Apply thin layer chromatography according to the General Rule 0502 and use polyamide as the coating substance and a mixture of acetone and a 4% solution of sodium acetate in water (3:10) as the mobile phase. Apply separately 1 μl of each of the test and reference drug solutions to the plate. After developing and removal of the plate, the solution is dried in air and examined under an ultraviolet light (254 nm). The fluorescent spots in the chromatogram obtained with the test solution should correspond in position and color to the spots in the chromatogram obtained with the reference drug solution.

Put 6 g of the contents of Tongxinluo capsules into 30 ml ethanol and the solution is filtered before ultrasonic treatment for 15 min. Evaporate the filtrate to dryness, wash the residue with 5 ml of ethyl ether for 2 min twice, discard the eluate, and expel ethyl ether. Dissolve the residue with l0 ml of water, apply it to a column packed with D101 macroporous resin (1.5 cm inner diameter, 12 cm long, pre-eluted successively with ethanol and water), elute with 150 ml water and discard the eluate. Elute again with 80 ml of 70% ethanol, collect the ethanol eluate and evaporate to dryness. Dissolve the residue in l ml ethanol to produce a test solution. Dissolve ginsenosides Rb_1_ CRS, Re CRS, Rg_1_ CRS in ethanol to prepare a reference solution containing 0.5 mg of each ingredient per ml. Apply thin layer chromatography according to General Rule 0502 and use silica gel G as the coating substance and the lower layer of a mixture of chloroform, methanol and water (13:7:2), kept below 10℃, as the mobile phase. Apply separately 5 μl of each of the test and reference drug solutions to the plate. After developing and removal of the plate, the solution is dried in air, sprayed with a 2% solution of vanillin in a mixture of sulfuric acid and ethanol, and heated at 105℃ until spots are clearly visualized. The spots in the chromatogram obtained with the test solution should correspond in position and color to the spots in the chromatogram obtained with the reference drug solution.

Put 0.5 g of the contents of Tongxinluo capsules into 5 ml petroleum ether (60-90℃) and shake the solution for 3 min. The supernatant is used as the test solution. Dissolve a mixture of borneol and isoborneol CRS in petroleum ether (60-90℃) to prepare a reference solution in 1 mg/ml. Apply thin layer chromatography according to the General Rule 0502, and use silica gel G as the coating substance and a mixture of toluene and acetone (10 :1) as the mobile phase. Apply separately 5 μl of each of the test and reference drug solutions to the plate. After developing and removal of the plate, the solution is dried in air, sprayed with a 2% solution of vanillin in sulfuric acid and ethanol, and heated until spots are clearly visualized. The spots in the chromatogram obtained with the test solution should correspond in position and color to the spots in the chromatogram obtained with the reference drug solution.

Put 3 g of the contents of Tongxinluo capsules into 20 ml ethanol, and the solution is filtered after ultrasonic treatment for 10 min. Evaporate the filtrate to dryness and dissolve the residue with 2 ml anhydrous ethanol to produce a test solution. Ultrasonication: Put 1 g of Olibanum reference drug into 5 ml anhydrous ethanol and the solution is filtered after ultrasonic treatment for 20 min. The filtrate is used as a reference drug solution. Apply thin layer chromatography according to the General Rule 0502, and use silica gel G as the coating substance and a mixture of petroleum ether (60-90℃) and ethyl ether (15:1) as the mobile phase. Apply separately 2-5 μl of the test solution and 5 μl of the reference drug solution to the plate. After developing and removal of the plate, the solution is dried in air, sprayed with a 2% solution of p-dimethylaminobenzaldehyde in a 10% solution of sulfuric acid in ethanol and heated at 105℃ until spots are clearly visualized. The spots in the chromatogram obtained with the test solution should correspond in position and color to the spots in the chromatogram obtained with the reference drug solution.

**Granularity**

Put 0.012 g of the contents of Tongxinluo capsules to 5 ml glycerol acetic acid test solution, which undergoes ultrasound treatment for 10 min. Shake to mix the solution well, drip a drop of the solution immediately onto a slide and cover with a coverslip (22 mm×22 mm). Prepare 5 slides in the same manner and measure the short diameters of the particles under a microscope with 200x magnification. Select randomly 5 fields in each slide with a total of 25 fields for 5 slides. Count the number of the particles with short diameters > 75 μm. The average number should be < 8 per field.

**Other requirements**

Comply with the general requirements for capsules (General Rule 0103).

**Content assay**

Apply high performance liquid chromatography according to General Rule 0512.

**Chromatographic system requirement:** Use the octadecylsilance bonded silica gel as the stationary phase and a mixture of acetonitrile and water (15:85) as the mobile phase. Set a detection wave length at 230 nm. The number of theoretical plates of the column should be ≥ 3,000, calculated with the reference to the peak of paeoniflorin.

**Reference solution:** Take 10 mg of paeoniflorin as a chemical reference substance, put it into a 100 ml of methanol and shake well. Take 5 ml of the solution, dilute it with 50 ml of 70% methanol and mix well to produce a reference solution containing 10 μg of paeoniflorin per ml.

**Test solution:** Put 0.25 g of the contents of Tongxinluo capsules into 25 ml of 70% methanol and the solution undergoes ultrasound treatment for 50 min. After cooling and weighing again, replenish the lost weight with 70% methanol, and shake the solution well before filtration. Take 10 ml of the filtrate, dilute it with 25 ml of 70% methanol, and shake the solution well before filtration. The filtrate is used as the test solution.

**Measurement:** Take 10 μl of each of the reference and the test solutions and put them into a HPLC instrument. The test solution sample should contain ≥ 0.30 mg paeoniflorin (C_23_ H_28_O_11_). ^1^

**Actions:** To tonify *qi*, activate blood, unblock the collaterals and relieve pain.

**Indications:** Tongxinluo capsules are indicated for angina pectoris caused by heart qi deficiency and static blood obstructing collaterals, manifested as chest compression, stabbing, gripping or localized chest pain, palpitation, spontaneous sweating, shortness of breath, tiredness, dark purple tongue with a blood stasis pattern, fine and rough pulse, or bound and intermittent pulse. Other indications include stroke belonging to qi deficiency and static blood obstructing collaterals, manifested as hemiplegia, hemianesthesia, deviated tongue and mouth, and sluggish speech.

**Recommended dosage:** Tongxinluo capsules should be administered orally, 2-4 capsules per time, 3 times daily. In this study, in order to make the compliance better, the dosage regimen was changed to 6 pills twice daily.

**Precautions and warnings:** Tongxinluo capsules are contraindicated in patients with hemorrhagic ulcer or with stroke caused by fire excess from yin deficiency, pregnant women, and women during menstruation.

**Drug specifications:** 0.26 g per capsule

**Storage:** Preserve in tightly closed containers

**The related research**

**Drugs introduction:**

Tongxinluo capsule, developed by Shijiazhuang Yiling Institute of Medicine, Hebei, China, is a capsule preparation of pure traditional Chinese medicine, and the formula of the capsule is designed according to the ancient theory on the pathogenesis and the long-term experience in the treatment of chest stuffiness and pains and stroke in the traditional Chinese medicine. By incorporating modern pharmacological studies and pharmaceutical technology, Tongxinluo capsule is produced to replenish qi, promote blood circulation, dredge meridians and relieve pain. Tongxinluo capsule in indicated in patients with angina pectoris caused by a deficiency of heart-qi and blockage of meridians by stagnated blood with manifestations of compression, fixed stabbing or anginal pain in the chest, palpitation, spontaneous sweating, shortness of breath, fatigue, dark purple or ecchymotic tongue, and thready or intermittent pulse. Tongxinluo capsule in also indicated in patients with stroke due to a deficiency of qi and stagnated blood blocking meridians presented with hemiplegia, hemianesthesia, deviated mouth and tongue and dysphasia.

**Drug ingredients and manufacture:**

The ingredients of Tongxinluo capsule include ginseng radix et rhizoma, hirudo, scorpio, paeoniae radix rubra, cicadae periostracum, eupolyphaga, scolopendra, santali albi lignum, dalbergiae odoriferae lignum, olibanum (processed), ziziphi pinosae semen (stir-baked), and borneolum syntheticum.

The manufacturing process of Tongxinluo capsule is as follows: 5 animal products hirudo, scorpio, cicadae periostracum, scolopendra and eupolyphaga are washed with water, dried up at low temperature, mixed with olibanum (processed), comminuted superfinely and sterilized with radiation for preparation. Volatile oil is extracted from santali albi lignum and dalbergiae odoriferae lignum, and the residue and aqueous solution are reserved for preparation. Ginseng radix et rhizoma is extracted twice by refluxing with ethanol solution and the extract solutions are mixed. Then, the ethanol is retrieved until alcohol odor disappears and the solution is reserved for preparation. The residue of ginseng radix et rhizoma, santali albi lignum and aalbergiae odoriferae lignum is combined with paeoniae radix rubra and ziziphi pinosae semen (stir-baked), decocted twice with water and the decoction is mixed and combined with the alcohol extract of ginseng radix et rhizome and the aqueous solution after oil is extracted from the santali albi lignum and dalbergiae odoriferae lignum. The mixed solution is further concentrated for preparation. The superfine powder and the extract is mixed well and granulated. Borneolum syntheticum is dissolved and made as volatile oil with ethanol, which is then sprayed into the above granules, mixed well and encapsulated for use.

**Toxicity test:**

Toxicity test using Tongxinluo capsule ultra-fine powder was carried out in rats for 6 months in order to ensure safe clinical applications. One hundred and sixty healthy SD rats with 70-90g weight, half male and half female, were randomly divided into four groups with 40 rats in each group: Tongxinluo small dose group, (1.62g crude drugs /kg, equivalent to 25 times of the clinical dose), Tongxinluo medium dose group (3.24g crude drugs /kg, equivalent to 50 times of the clinical dose), Tongxinluo large dose group (6.48g crude drugs /kg, equivalent to100 times of the clinical dose) and control group. Tongxinluo capsule ultra-fine powder was administrated intragastrically in rats for 26 weeks. General examination, laboratory assays and histopathological studies were performed in 10, 20 and 10 rats in each group in 13th, 26th and 28th week after treatment, respectively. The results showed that there was no apparent difference between three treatment groups and control group in the coat color, skin, behavior, secretion and excreta, and body weight and food intake. Similarly, there was no significant difference between three treatment groups and control group in coagulation time, hemoglobin level, erythrocyte, leukocyte and platelet counts, serum levels of ALT, AST and AKP, levels of blood sugar, total bilirubin, urea nitrogen, creatinine, total protein, albumin, and total cholesterol, and histopathological observations of the heart, liver, spleen, lung, kidney, brain, stomach, duodenum, pancreas, adrenal, thymus, thyroid, prostate, testis, ovary and uterus.

**Clinical overview**

1. **Efficacy and safety of Tongxinluo capsules made of ordinary powder in patients with angina pectoris**

In the original clinical report submitted to the State Food and Drug Administration in 1996，a multicenter, randomized, single-blind and parallel-group study was performed to assess the therapeutic effect and safety of Tongxinluo capsules made of ordinary powder in comparison to an approved traditional Chinese medication in patients with angina pectoris. A total of 492 patients were divided into Tongxinluo group (4 capsules each time, three times daily) and control group receiving an approved traditional Chinese medication, and the study duration was 28 days. The following parameters were assessed: 1. Symptomatic relief of angina pectoris which was classified as (1) highly effective: symptoms completely or basically disappear with nitroglycerin withdrawal; (2) effective: frequency, severity and duration of angina pectoris were significantly alleviated with the dose of nitroglycerin reduced by more than 50%; (3) ineffective: Symptoms were basically unchanged or aggravated after treatment with the dose of nitroglycerin increased; 2. Electrocardiogram improvement which was classified as (1) highly effective: electrocardiogram was recovered to normal or basically normal; (2) effective: the magnitude of ST segment depression was reduced by 0.05mV after treatment but did not return to a normal level; (3) ineffective: electrocardiogram was basically unchanged or aggravated after treatment; 3. Parameters of safety: Routine assays of blood, urine and stool, hepatic and renal functions and adverse events were evaluated.

The results showed that for symptomatic relief, the cases (percentage) showing highly effective efficacy in the Tongxinluo and control groups were 214 (62.57%) and 47 (31.33%), respectively (P<0.01). Specifically, Tongxinluo group showed reduced frequency and duration of angina in comparison with the control group (P<0.05) and the percentage of withdrawal and dose reduction of nitroglycerin in the Tongxinluo and control groups were 94.44% and 73.68%, respectively (P<0.01). For electrocardiogram improvement, the cases (percentage) showing highly effective efficacy in the Tongxinluo and control groups were 109 (31.87%) and 27 (18.00%), respectively (P<0.01). As for the safety assessment, there was no statistical significance between the two groups in any laboratory parameters measured (P>0.05). In summary, the total effective rate and highly effective rate were 91.23% and 51.46% in the Tongxinluo group, and 68.67% and 28% in the control group, respectively. It was concluded that Tongxinluo treatment was significantly better than the approved traditional Chinese medication in improving the symptoms and electrocardiogram of patients with angina pectoris. ^2^

1. **Comparison of Tongxinluo capsules made of common and ultrafine powders and with different doses in patients with angina pectoris**

In the original clinical report submitted to the State Food and Drug Administration in 2005，the therapeutic effect and safety of Tongxinluo capsules made of common and ultrafine powder and with different doses were compared in patients with angina pectoris. A multicenter, randomized, double blind, parallel-group and placebo-controlled study was conducted and 288 patients with angina pectoris were divided into two groups: the test group with Tongxinluo capsules made of ultrafine powder and the control group with Tongxinluo capsules made of ordinary powder. Each group was further divided into two subgroups with 72 cases in each subgroup: one subgroup receiving 2 capsules each time, three times daily and the other receiving 4 capsules each time, three times daily. The study duration was 28 days. The following parameters were assessed: 1. Symptomatic relief of angina pectoris which was classified as (1) highly effective: symptoms completely or basically disappear with nitroglycerin withdrawal; (2) effective: frequency, severity and duration of angina pectoris were significantly alleviated with the dose of nitroglycerin reduced by more than 50%; (3) ineffective: Symptoms were basically unchanged or aggravated after treatment with the dose of nitroglycerin increased; 2. Electrocardiogram improvement which was classified as (1) highly effective: electrocardiogram was recovered to normal or basically normal; (2) effective: the magnitude of ST segment depression was reduced by 0.05mV after treatment but did not return to a normal level; (3) ineffective: electrocardiogram was basically unchanged or aggravated after treatment; 3. Parameters of safety: Routine assays of blood, urine and stool, hepatic and renal functions and adverse events were evaluated.

The results showed that for symptomatic relief, the cases (percentage) showing highly effective efficacy in the test and control groups taking 2 capsules each time were 35/68 (51.5%) and 32/69 (46.4%), and the cases (percentage) showing highly effective or effective efficacy in these patients were 60/68 (88.2%) and 59/69 (85.5%), respectively, with no significant difference between the two groups (P>0.05). By comparison, the cases (percentage) showing highly effective efficacy in the test and control groups taking 4 capsules each time were 44/69 (63.8%) and 42/70 (60.0%), and the cases (percentage) showing highly effective or effective efficacy were 66/69 (95.7%) and 63/70 (90.0%), respectively, with no significant difference between the two groups (P>0.05). For electrocardiogram improvement, the cases (percentage) showing highly effective efficacy in the test and control groups taking 2 capsules each time were 17/68 (25.0%) and 15/69 (21.7%), and the cases (percentage) showing highly effective or effective efficacy in these patients were 46/68 (63.2%) and 42/69 (60.9%), respectively, with no significant difference between the two groups (P>0.05). By comparison, the cases (percentage) showing highly effective efficacy in the test and control groups taking 4 capsules each time were 21/69 (30.4%) and 18/70 (25.7%), and the cases (percentage) showing highly effective or effective efficacy in these patients were 49/69 (71.0%) and 47/70 (67.1%), respectively, with no significant difference between the two group (P>0.05). Although the therapeutic effects of large dose of Tongxinluo capsules seemed to be superior to those of low dose of Tongxinluo capsules, the difference was not significant, probably due to a small sample size. As for the safety assessment, there was no significant significance between the two groups in any laboratory measurements (P>0.05). The incidence of adverse effect was 0% in the test group and 2.08% in the control group, and all symptoms related to treatment was gastric indisposition noted in subgroups taking 4 capsules each time made of ordinary powder. Thus, compared with Tongxinluo capsules made of ordinary powder, Tongxinluo capsules made of ultrafine powder reduced the incidence of gastric indisposition and had a better safety profile. It was concluded that in patients with angina pectoris, Tongxinluo capsules made of ultrafine powder had a similar therapeutic effect to but a better safety profile than Tongxinluo capsules made of ordinary powder. Thus, it was recommended that the routine dose for Tongxinluo capsules made of ultrafine powder is four capsules each time, three times daily.

**3.** **Efficacy and safety of Tongxinluo capsules made of ordinary powder in patients with stroke**

In the original clinical report submitted to the State Food and Drug Administration in 1998，a multicenter, randomized and single-blind study was performed to evaluate the therapeutic effects of Tongxinluo capsule made of ordinary powder in patients with ischemic stroke. A total of 125 patients with ischemic stroke at recovery stage (2 weeks to 6 months after stroke) were randomly divided into Tongxinluo and control groups, who on the basis of standard treatment, received Tongxinluo capsules made of ordinary powder (4 capsules each time, 3 times daily) and placebo, respectively, for 4 weeks. Patients’ neurological functional defects were scaled as 8 ~ 30 points and their ability to conduct daily activity was scaled as 2 ~ 5 grades. The efficacy was assessed by a scoring method based on the following formula: therapeutic percentage score= [(pre-therapy scores- post-treatment scores)/pre-therapy scores] x 100%, and the derived scores were divided into four classes: essential recovery: ≥85%, excellence: ≥50%, effectiveness: ≥20% and ineffectiveness: <20%. The results showed that in the Tongxinluo group, 15.4% of the patients exhibited essentially recovery, 47.4% excellence and 28.2% effectiveness, resulting in a 91.3% of total effectiveness rate. By comparison, in the control group, 10.6% patints exhibited essentially recovery, 27.7% excellence and 42.6% effectiveness, resulting in an 80.9% of total effectiveness rate. These results indicated that Tongxinluo treatment in addition to standard treatment further improved the functional rehabilitation in patients with ischemic stroke.

**4. Comparison of Tongxinluo capsules made of common and ultrafine powders in patients with stroke**

To assess the efficacy and safety of Tongxinluo capsules made of common and ultrafine powder in patients with ischemic stroke, a multicenter, randomized, double-blind and parallel-group study was performed in 2005. A total of 144 patients with ischemic stroke at recovery stage (2 weeks to 3 months after stroke) were divided randomly into test and control groups who received Tongxinluo capsules made of ultrafine and ordinary powder, respectively, at a dose of 4 capsules each time, 3 times daily for 4 weeks. Patients’ neurological function defect was scaled as 8 ~ 30 points and their ability to conduct daily activity was scaled as 2 ~ 5 grades. The efficacy was assessed by a scoring method based on the following formula: therapeutic percentage score= [(pre-therapy scores- post-treatment scores)/pre-therapy scores] x 100%, and the derived scores were divided into four classes: essential recovery: ≥85%, excellence: ≥50%, effectiveness: ≥20% and ineffectiveness: <20%. Drug safety and adverse events were also assessed. The results revealed that in the test group, 13.2% of the patients exhibited essential recovery and 44.1% excellence, resulting in a total effectiveness rate of 91.2%. By comparison, in the control group, 11.3% of the patients showed essential recovery and 42.3% excellence, resulting in 87.3% total effectiveness rate. There were no significant differences in clinical efficacy between the two groups. The incidence of adverse events was 2.8% and 6.9% in the test and control groups, respectively. Meanwhile, the incidence of drug-related side effects was 1.4% and 2.8% in the test and control groups, respectively. These results indicated that Tongxinluo capsules made of ultrafine powder exhibited a better safety profile than and similar therapeutic efficacy to Tongxinluo capsules made of ordinary powder in the treatment of patients with ischemic stroke. ^3^

**References**

1. **Chinese Pharmacopoeia Commission.** [Pharmacopoeia of the People’s Republic of China]. Beijing: People’s Medical Publishing House; 2005.
2. **Guicheng Xu, Ronglin Gao, Yiling Wu, Junling Liu, Xuedong Gao, Hui Li.** Clinical study on Tongxinluo Capsule in treatment of patients with angina pectoris caused by coronary heart. *Chinese Journal of Integrated Traditional and Western Medicine* 1997; 17: 414–416.
3. **Yiling Wu, Tao Li, Yan Li, et al.** Clinical study of super crush-run Tongxinluo capsule on treatment of stroke. *China Journal of Chinese Materia Medica* 2007; 32: 1928–1931.

**Supplementary appendix IV: The randomization and blinding**

Randomization:

The investigators assign the informed patients a screen code, then determine the patient eligibility and register the eligible patient in the IWRS (Interactive Web Response System, Version 3.1, Bioguider Medical Technology Co. Ltd, Shanghai) which will assign a unique randomization code. The randomization code and package code were generated by the biostatistician (Center for Drug Clinical Research, Shanghai University of Traditional Chinese Medicine, Shanghai) who was irrelevant to the trial using DAS 2.1.1 software package. Randomization was performed in block of six (3：3), the randomization data can be supplied when necessary. According the randomization code, the patients were allocated to either TXL group or placebo group at 1:1 ratio in permuted stacked blocks stratified by study center. The randomization code and package code were imported into the IWRS by random administrator (Maosheng Zhou). This scheme will be generated and maintained by one of the co-principal investigators not directly involved with patient enrolment. Allocation will be concealed using the consecutively, numbered, sealed opaque envelope technique. As such it will not be possible for the treating clinicians, or other study investigators to know the treatment group assignment prior to randomization. Only in the event of a serious adverse experience, when the investigator deems that the subject cannot be adequately treated without knowing the identity of the study medication, may the medication code be broken for a particular subject. All analysis was based on the intention-to-treat principle. Hence, the full analysis set was defined as all patients who were randomly assigned.

Trial blinding:

The placebo capsules used in this trial were identically sized capsules filled with lactose powder, and colored to match the Tongxinluo capsules without any marking. Statistician generated random number table by SAS software, pharmacists gave numbers to investigational product or placebo according to random number table, and pharmacists, investigators, nurses, lab personnel will be blinded to study group assignment, and all safety outcomes will be objectively reviewed in a blinded fashion.

**Supplementary appendix V: Instructions for** **carotid artery ultrasonography examination and image analyses**

**Requirements for sonographers and instruments**

In an attempt to standardize image acquisitions and measurements, all sonographers from each participating center received intensive training in carotid artery ultrasound investigation in the core echo laboratory, Shandong University Qilu Hospital, and two experienced sonographers from the core echo laboratory paid a visit to each participating center to guide and supervise image acquisition per the study protocol. Prior to participant enrollment, each laboratory was required to record a full set of images from a number of sample cases that were sent to the core echo laboratory for quality control. Expert feedback on these recordings was returned to each participating laboratory for improvement of image acquisition. To reduce the image variability among different vendors, two brands of ultrasonic systems IE 33 (Phillips Medical Systems) and VIVID E9 (GE Healthcare) were used in all image acquisitions and analyses.

**Methods of image acquisition**

The carotid artery ultrasonic examination was performed at baseline, and 12 and 24 months after enrollment in the study. To fully expose the patient’s neck, all patients assumed a supine position with the shoulders padded high and head tilted back. A high frequency probe（7-11MHz）connected to an ultrasonic system was used to scan bilateral carotid arteries and an electrocardiogram was simultaneously recorded. The probe was first put on patient’s left or right upper concave of clavicle, and moved from the proximal end of the carotid artery to the carotid bifurcation and internal carotid artery in sequence in the longitudinal axis views, ensuring the region of interest from the carotid artery 3cm proximal to the bifurcation to the internal carotid artery 1 cm distal to the bifurcation was fully scanned. The optimal image should display the tip of the blood flow divider or the “Y” appearance of the common artery and internal and external carotid arteries. The anterior and posterior wall images should be obtained from bilateral carotid arteries. Thereafter, the probe was rotated for 90° to derive the cross-sectional images of the carotid artery and scanned from the proximal end of the carotid artery to the carotid bifurcation and internal carotid artery in sequence in the short axis views, ensuring that the vascular intima, media and adventitia, and the location, shape and echogenicity of the carotid plaque were clearly visualized. For the repeatability and reproducibility, the images of each follow-up must be based on the previous. The carotid artery images of at least 5 cardiac cycles are recorded and digitally stored, which is sent to the core echo laboratory for further analyses.

**1. Measurement of the carotid IMT**

The digitized still images from an electrocardiographically defined diastolic frame were analyzed offline, and all data were measured three times to derive the calculated mean value. As per the study protocol, the mean IMT in the long-axis view of the carotid artery was obtained from the following 12 points of bilateral carotid arteries:

(1) The near and far walls of bilateral common carotid arteries 2cm proximal to the tip of the blood flow divider;

(2) The near and far walls of bilateral common carotid arteries 1cm proximal to the tip of the blood flow divider;

(3) The near and far walls of bilateral internal carotid arteries bifurcation 1cm to the tip of the blood flow divider.

If there was a plaque located at these 12 sites, it was included in the IMT measurement. (Figure S2)

**2. Measurement of the carotid plaque area**

The definition of a plaque is a focal thickening the carotid IMT ≥1.2 mm, which is ≥ 50% thicker than the IMT proximal or distal to the plaque. After a carotid plaque was detected, the maximal plaque area in the long-axis view was measured by manually tracing the thickened intima in the lumen and the interface between the adventitia and the media along the wall (Figure S3). The maximal plaque area in the short-axis view was derived by subtracting the lumen area from the external elastic membrane area traced manually (Figure S4).

**3. Measurement of vascular remodeling index**

Once a carotid plaque was detected, the locations 5 mm proximal and distal to the plaque were chosen as the reference sites (Figure S5). The carotid artery diameter from the external elastic membrane of the near wall to that of the far wall was measured at the maximal plaque site and two reference sites and the vascular remodeling index was derived by calculating the ratio of the vessel diameter at the plaque site to the average of the vessel diameters at two reference sites. A vascular remodeling index >1.05 was considered positive remodeling, <0.95 negative remodeling and 0.95-1.05 no remodeling.

**Image storage and analyses**

All ultrasound images were stored in the DICOM format and recorded on a DCD disk. During this process, all information of research institutions, subjects, and sonographers was digitally masked from the images, which were then randomized and sent to the core echo laboratory for image analysis by two experienced physicians who used commercially available software (TomTec Image-com). All sonographers and reading physicians were completely blinded to the group of enrolled participants. Rejected images due to incompleteness and poor quality were further checked by the director of the core echo laboratory.

All ultrasonic data were measured 3 times and the average was adopted. To examine the reproducibility of ultrasonic measurements, three key variables including mean IMT, plaque area and vascular remodeling index, were re-measured in 50 randomly selected subjects by two independent investigators. Inter-observer variability was assessed by two investigators and intra-observer variability was assessed by one investigator at different times. Bland-Altman plots were used to analyze the inter- and intra-observer variability and interclass correlation coefficients (ICCs) were calculated.**Supplementary appendix VI: Supplementary figures**

**Supplementary Fig. S1 online. Scheduled Follow-up Visits throughout the Study**


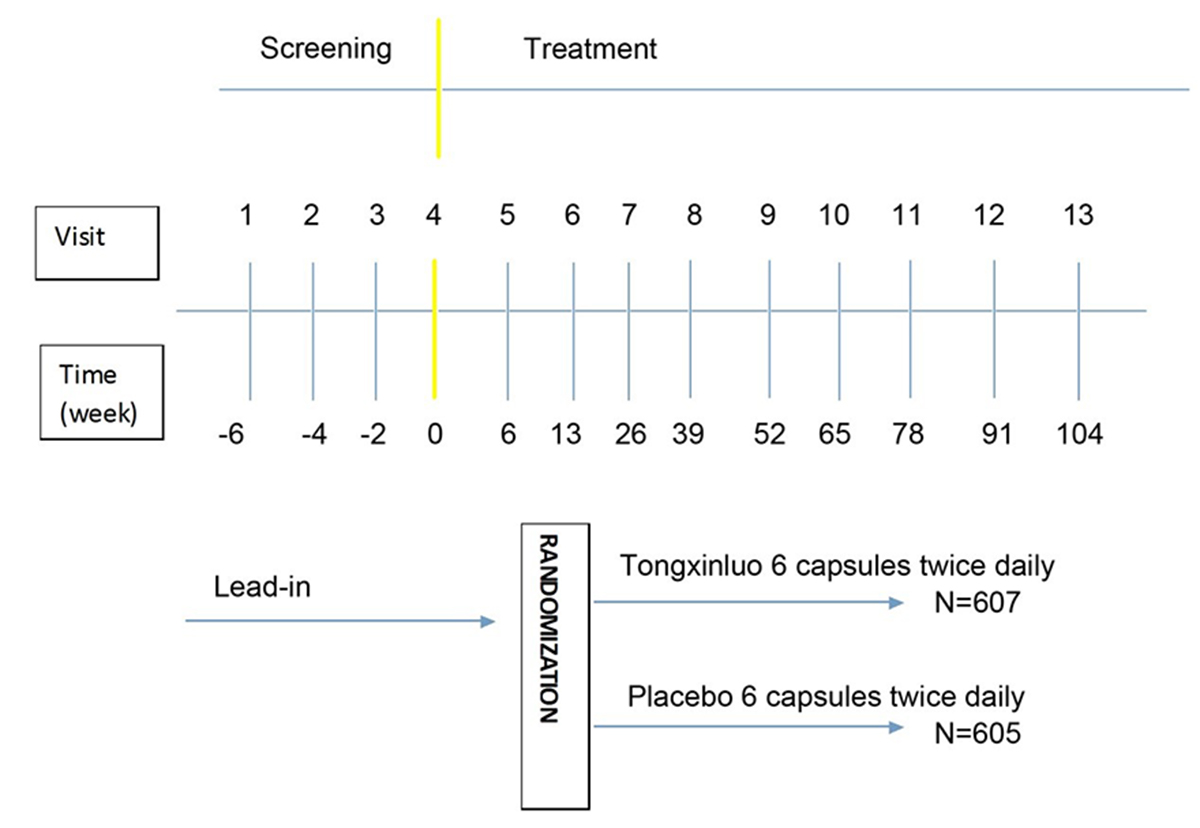


Eligible patients were randomized blindly at a 1:1 ratio to receive 6 capsules of Tongxinluo or 6 capsules of placebo, twice daily for 24 months.

**Supplementary Fig. S2 online. Measurement of the Carotid Intima-media Thickness (IMT)**


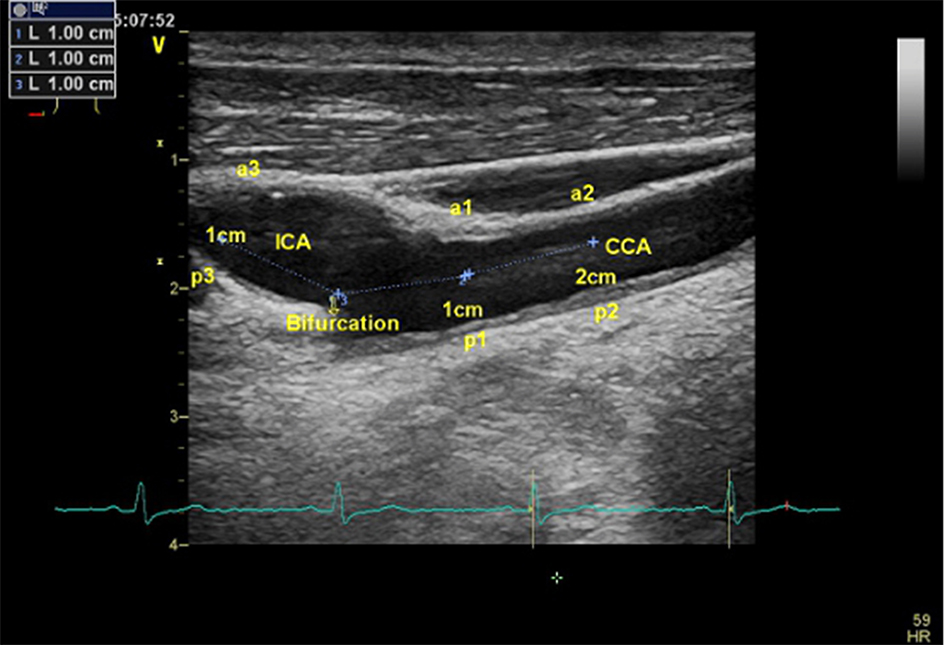


Letters a1, a2 and a3 denoted the sites 1cm and 2cm proximal to the carotid bifurcation in the near wall of the common carotid artery and 1 cm distal to the carotid bifurcation in the near wall of the internal carotid artery, respectively. Letters b1, b2 and b3 denoted the sites 1cm and 2 cm proximal to the carotid bifurcation in the far wall of the common carotid artery and 1 cm distal to the carotid bifurcation in the far wall of the internal carotid artery, respectively.

**Supplementary Fig. S3 online. Measurement of the Carotid Plaque in the Long-axis View**


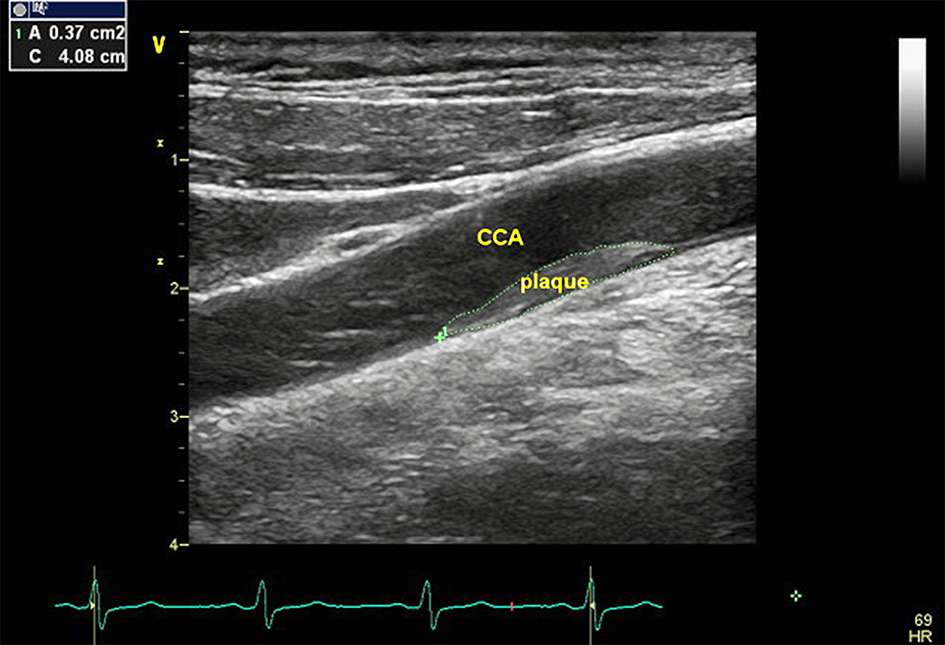


The maximal plaque area in the long-axis view was measured by manually tracing the thickened intima in the lumen and the interface between the adventitia and the media along the wall.

**Supplementary Fig. S4 online. Measurement of the Carotid Plaque in the Short-axis View**


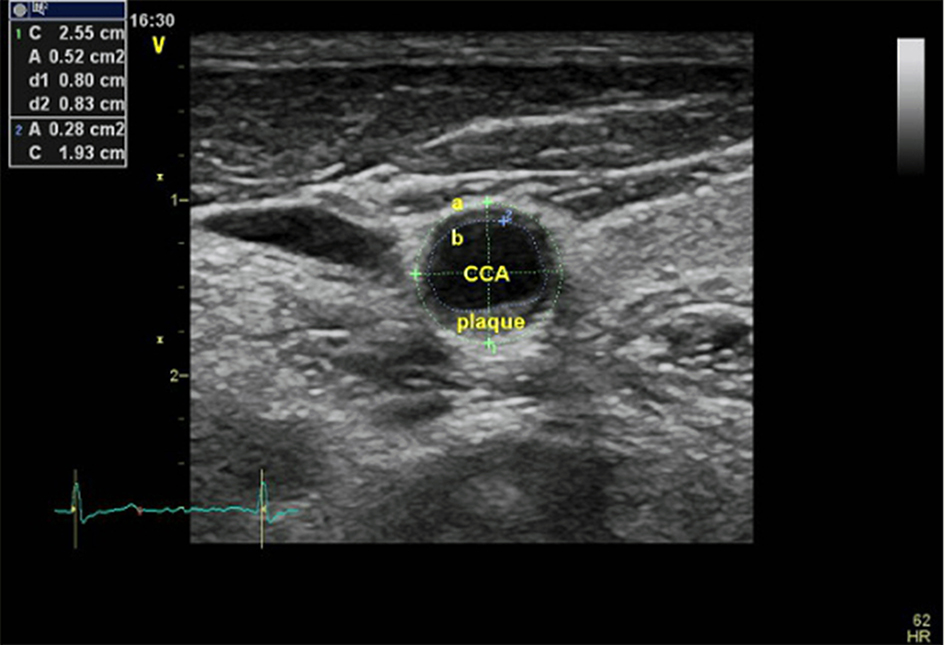


Letters a and b denoted the area of external elastic membrane (i.e., the cross-sectional area between tunica elastica and adventitia) and the area of the vascular lumen, respectively. The plaque area was defined as the difference between a and b.

**Supplementary Fig. S5 online. Measurement of Vascular Remodeling Index**


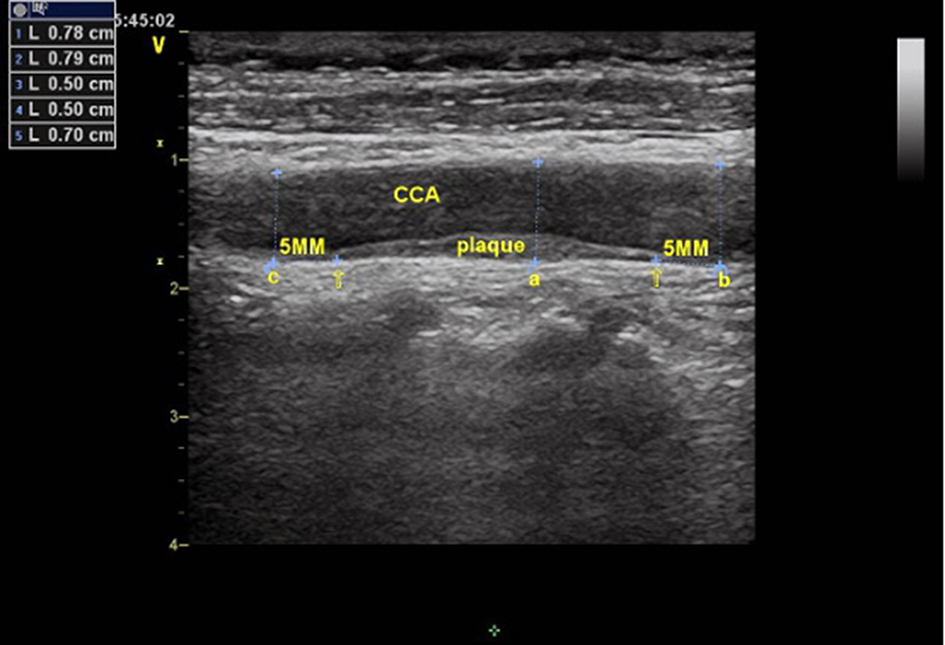


Letters a, b, and c indicated the largest thickness of the carotid plaque and the reference positions 5 mm proximal and 5 mm distal to the plaque, respectively. The vertical distance between the adventitia of the near and far walls at the plaque and two reference sites was used to calculate vascular remodeling index.

**Supplementary appendix VII: Definition of major cardiovascular events**

Major cardiovascular events were defined as the first occurrence of unstable angina pectoris, nonfatal myocardial infarction, cardiac death, coronary revascularization, stroke and death from patient enrollment to the last follow-up. Unstable angina pectoris was defined as a clinical syndrome between stable angina pectoris and acute myocardial infarction, including first onset angina, aggravating effort angina, resting angina with electrocardiographic evidence of myocardial ischemia and post-myocardial infarction angina. Nonfatal myocardial infarction included ST-elevated myocardial infarction (ST-segment elevation >0.5 mv) and non-ST-elevated myocardial infarction (ST-segment depression ≥ 0.1mv), with clinical manifestation of unstable angina pectoris and typical changes of serum levels of troponin I (cTnI), which reaches the maximal serum level after 11-24 hr and returns to the normal serum level after 7-10 days, or troponin T(cTnT), which increases to the maximal serum level after 24-48 hr and decreases to the normal serum level after 10-14 days, or creatine kinase isoenzyme (CK-MB), which increases to the maximal serum level after 16-24 hr and decreases to normal levels after 3-4 days. In patients with ST-elevated myocardial infarction, electrocardiograms showed a convex ST-segment elevation and Q waves >30 ms in duration and >25% of the R-wave amplitude in depth in leads facing the necrotic area. In patients with non-ST-elevated myocardial infarction, electrocardiograms showed a temporary ST-segment depression (≥ 0.1 mv). Cardiac death was defined as death caused by cardiac diseases. Coronary revascularization referred to thrombolysis after acute myocardial infarction, coronary artery intervention and coronary artery bypass grafting in enrolled patients at least 30 days after randomization. Stroke was defined as transient ischemic attack and cerebral infarction.

**Supplementary appendix VIII: Research evidence in context**

**Evidence before this study**

We searched MEDLINE, the China National Knowledge Internet, China Biology Medicine Database, VIP Database and Wanfang Database for relevant randomized, placebo-controlled, double-blind clinical trials. We considered publications in either English or Chinese. Literatures were searched in MEDLINE with the terms (“traditional Chinese medicine” OR “traditional Chinese medication” OR “TCM”) AND (“intima-media thickness” OR “IMT”) AND “randomized” AND “double-blind”, with no restriction on subheadings. We adapted search terms for use in databases and search engines, and checked the reference lists of retrieved articles for further potential citations. We primarily focused on articles published prior to December 1, 2008 before start of the CAPITAL study.

After screening, only two articles from the same research group, both in Chinese, were eligible for inclusion. One study enrolled 79 patients with diabetes and the other enrolled 88 patients with diabetes and hypertension. These patients were randomly divided into two groups: TCM group using ingredients including *Rehmannia glutinosa (Gaert.) Libosch. ex Fisch. et Mey.*, *Cornus officinalis* Sieb. et Zucc., *Cortex Moutan*, *Dioscorea opposite*, *Wolfiporia cocos*, *Alisma plantago-aquatica Linn.*, and *Folium Ginkgo*, and control group using placebo, in addition to western medicine treatment. The primary end-points were intima-media thickness (IMT) of the common or right carotid artery after18 month follow-up. The results showed that TCM treatment significantly inhibited the progression of IMT in comparison to placebo treatment. However, the sample size in these two studies was too small for the results to be conclusive.

**Added value of this study**

Previous studies on the effect of traditional Chinese medicine on the progression of IMT in patients with carotid atherosclerosis were promising but limited by small sample size. The CAPITAL study demonstrated that in a large cohort of patients with subclinical carotid atherosclerosis, in addition to current routine therapy, administration of TXL capsules for 24 months strikingly halted the progression of the mean IMT and maximal plaque area of the carotid artery. In addition, TXL treatment significantly attenuated positive vascular remodeling of the carotid artery and reduced the incidence of unstable angina. Thus, the CAPITAL study is the first to provide convincing evidence of the added value of traditional Chinese medicine in treatment of atherosclerosis.

**Implications of all the available evidence**

A number of observational studies showed that TXL capsule lowered serum lipid levels, improved angina pectoris and reduced the incidence of restenosis in patients with coronary heart disease (References 6-8 in the manuscript). Early clinical trials compared lovastatin or rosuvastatin to placebo for their efficacy on the progression of the carotid mean IMT and found that both stains had a superior effect (References 13-14 in the manuscript). However, when combined with simvastatin, ezetimibe was not superior to placebo in affecting progression of carotid mean IMT in patients with familial hypercholesterolemia (Reference 15 in the manuscript). In the present study, we compared TXL to placebo for efficacy on progression of the carotid mean IMT, in addition to current routine therapies including aspirin, statins, ACEI/ARBs and calcium antagonists, and found that TXL had a positive effect. The Asymptomatic Carotid Artery Progression Study (ACAPS) found that a carotid IMT reduction of 0.009 mm/year was associated with a significant reduction in major cardiovascular events. In the METEOR trial, progression of the carotid mean IMT was −0.0014 and 0.0131 mm/year with rosuvastatin and placebo treatment, respectively. Our study showed progression of the carotid mean IMT of -0.00095 and 0.01312 mm/year for the TXL and placebo groups, respectively, with a significant reduction in incidence of unstable angina in the TXL versus placebo group at 24 months. These results were better than those of the ACAPS and METEOR trials because in the latter two trials, no anti-atherosclerotic medications were given in the placebo groups. The annual reduction in mean IMT was greater for patients receiving both statins and TXL than TXL alone. In view of the fact that many patients remain at high residual risk for cardiovascular events even after intensive statin treatment and side effects of statin administration may be more common in Chinese than Western population, the added value of TXL in the treatment of atherosclerosis has paramount clinical significance.

**Supplementary appendix IX:**

**Table S1 Incidence of major cardiovascular events in two groups of patients**

| **Events** | **Tongxinluo group**  **（n=607）**  **no**. **(%)** | **Placebo group**  **（n=605） no**. **(%)** | **P Values** |
| --- | --- | --- | --- |
| Unstable angina pectoris | 38 (6.3%) | 65 (10.7%) | 0.005 |
| Nonfatal myocardial infarction | 1 (0.2%) | 5 (0.8%) | 0.218 |
| Cardiac death | 0 (0.0) | 0 (0.0) | 1.000 |
| Coronary revascularization | 3 (0.5%) | 4 (0.7%) | 0.997 |
| Stroke | 9 (1.5%) | 11 (1.8%) | 0.647 |
| Death | 0 (0.0) | 3 (0.5%) | 0.247 |
| Total | 47 (7.7%) | 80 (13.2%) | 0.002 |

Major cardiovascular events were reported in 47 patients (7.7%) of the TXL group and in 80 patients (13.2%) of the placebo group. The occurrence of unstable angina pectoris was lower in the TXL than placebo group (P=0.005)

**Table S2 Major adverse reactions in two groups of patients**

| **Adverse reactions** | **Tongxinluo (n=604)**  **no. (%)** | **Placebo(n=605)**  **no. (%)** | **P Values** |
| --- | --- | --- | --- |
| Hepatic insufficiency | 7 (1.2) | 5 (0.8) | 0.560 |
| Renal insufficiency | 1 (0.2) | 2 (0.3) | 1.000 |
| Headache | 10 (1.7) | 11 (1.8) | 0.829 |
| Stomach discomfort | 24 (4.0) | 14 (2.3) | 0.098 |
| Abdominal pain or diarrhea | 4 (0.7) | 6 (1.0) | 0.753 |
| Bleeding or delayed PT | 8 (1.3) | 2 (0.3) | 0.112 |
| Allergic rash or asthma | 1 (0.2) | 1 (0.2) | 1.000 |
| Mental disorders | 0 (0.0) | 1 (0.2) | 1.000 |
| Insomnia | 0 (0.0) | 3 (0.5) | 0.248 |
| Total | 55 (9.1) | 45 (7.4) | 0.292 |

The TXL and placebo groups did not differ in terms of adverse reactions reported. Adverse related to treatment occurred in 55/604 patients (9.1%) and 45/605 patients (7.4%) in the TXL and placebo groups, respectively (P=0.292).
